# The crystal structures of three disordered 2-substituted benzimidazole esters

**DOI:** 10.1107/S2056989021003364

**Published:** 2021-04-09

**Authors:** Chayanna Harish Chinthal, Hemmige S. Yathirajan, Nagaraja Manju, Balakrishna Kalluraya, Sabine Foro, Christopher Glidewell

**Affiliations:** a Department of Studies in Chemistry, University of Mysore, Manasagangotri, Mysuru-570 006, India; b Department of Studies in Chemistry, Mangalore University, Mangalagangotri, Mangalore-574199, India; c Institute of Materials Science, Darmstadt University of Technology, Alarich-Weiss-Strasse 2, D-64287 Darmstadt, Germany; d School of Chemistry, University of St Andrews, St Andrews, Fife KY16 9ST, UK

**Keywords:** heterocyclic compounds, benzimidazoles, crystal structure, disorder, hydrogen bonding, supra­molecular assembly

## Abstract

The crystal structures of three benzimidazole esters containing aryl or heterocyclic substituents at position 2 are reported, and all three exhibit disorder of mol­ecular entities. In ethyl 1-methyl-2-[4-(prop-2-yn­oxy)phen­yl]-1*H*-benzimidazole-5-carboxyl­ate, C_20_H_18_N_2_O_3_, (I), the prop-2-yn-1-oxyphenyl unit is disordered over two sets of atomic sites having effectively equal occupancies, 0.506 (5) and 0.494 (5). The propyl substituent in ethyl 1-propyl-2-(pyren-1-yl)-1*H*-benzimidazole-5-carboxyl­ate, C_29_H_24_N_2_O_2_, (II), is disordered over two sets of atomic sites having occupancies 0.601 (8) and 0.399 (8), and the ester unit in ethyl 1-methyl-2-(5-chloro-3-methyl-1-phenyl-1*H*-pyrazol-4-yl)-1*H*-benzimidazole-5-carboxyl­ate, C_21_H_19_ClN_4_O_2_ (III), is disordered over two sets of atomic sites having occupancies 0.645 (7) and 0.355 (7). In each of the C—H⋯π(arene) hydrogen bonds in (I), the donor and acceptor form parts of different disorder components, so that no continuous aggregation is possible. The mol­ecules of (II) are linked by a single C—H⋯O hydrogen bond into *C*(10) chains, which are linked into sheets by a π–π stacking inter­action, whereas those of (III) are just linked into *C*(13) chains, again by a single C—H⋯O hydrogen bond. Comparisons are made with the structures of some related compounds.

## Abstract 2

Three 2-substituted benzimidazole esters each exhibit different types of mol­ecular disorder and different patterns of supra­molecular assembly.

## Chemical context   

**Figure 1:**
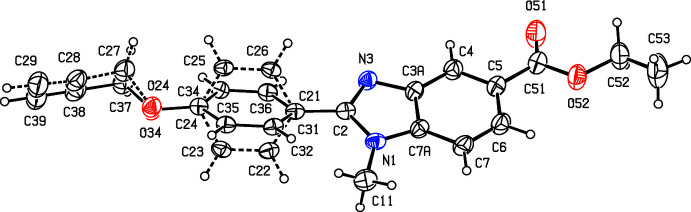
The mol­ecular structure of compound (I) showing the atom-labelling scheme and the disorder. The major disorder form is drawn using full lines and the minor disorder component is drawn using broken lines. Displacement ellipsoids are drawn at the 30% probability level.

**Figure 2:**
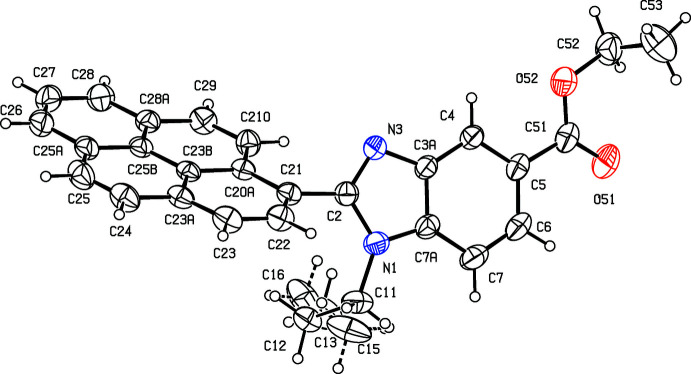
The mol­ecular structure of compound (II) showing the atom-labelling scheme and the disorder. The major disorder form is drawn using full lines and the minor disorder component is drawn using broken lines. Displacement ellipsoids are drawn at the 30% probability level.

The use of compounds containing the benzo[*d*]imidazole unit as chemotherapeutic agents having anti­microbial, anti­parasitic, anti­tumour and anti­viral activity has been comprehensively reviewed (Boiani & Gonzalez, 2005 ▸). In particular, 2-substituted benzo[*d*]imidazoles have recently been evaluated for their anti­microbial and anti­oxidant activity (Zhou *et al.*, 2013 ▸; Bektaş *et al.*, 2020 ▸). With these considerations in mind, we have synthesized some new 2-substituted benzo[*d*]imidazoles and here we report the structures of two new benzimidazole esters, namely ethyl 1-methyl-2-[4-(prop-2-yn­oxy)phen­yl]-1*H*-benzimidazole-5-carboxyl­ate (I) (Fig. 1 ▸) and ethyl 1-propyl-2-(pyren-1-yl)-1*H*-benzimidazole-5-carboxyl­ate (II) (Fig. 2 ▸) carrying aromatic substituents at position 2 of the heterocyclic ring.

**Figure 3:**
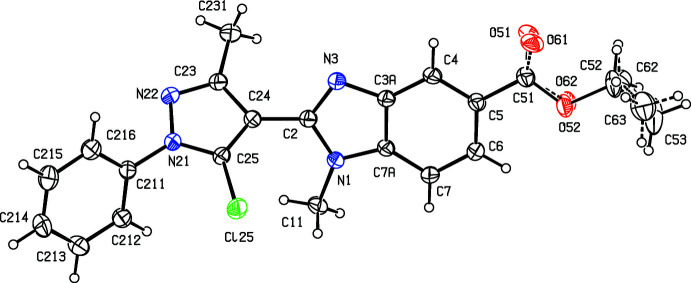
The mol­ecular structure of compound (III) showing the atom-labelling scheme and the disorder. The major disorder form is drawn using full lines and the minor disorder component is drawn using broken lines. Displacement ellipsoids are drawn at the 30% probability level.

The structure of the related compound ethyl 1-methyl-2-(5-chloro-3-methyl-1-phenyl-1*H*-pyrazol-4-yl)-1*H*-benzimidazole-5-carboxyl­ate (III) (Fig. 3 ▸) was reported recently (Manju *et al.*, 2018 ▸), but the reported refinement was based on a rather unusual disorder model, in which only some of the atoms in the ester function, namely the methyl­ene group and the H atoms of the methyl group, were described as disordered over two sets of atomic sites having occupancies 0.719 (14) and 0.281 (14), but with all other components of this substituent fully ordered. This model leads to some unexpected distances within the eth­oxy unit, O—C =1.480 (4) and 1.618 (13) Å and C—C = 1.274 (6) and 1.295 (10) Å, which in turn cast doubt on the correctness of the disorder model. Accordingly, we have taken the opportunity to collect a new, and rather better data set for compound (III) [4250 reflections with *R*_int_ = 0.0126 as against 4010 reflections with *R*_int_ = 0.0418 (Manju *et al.*, 2018 ▸)] and, using a more realistic disorder model, we have refined the structure of (III) to *R*_1_ = 0.0395 as against a value of 0.0526 (Manju *et al.*, 2018 ▸).
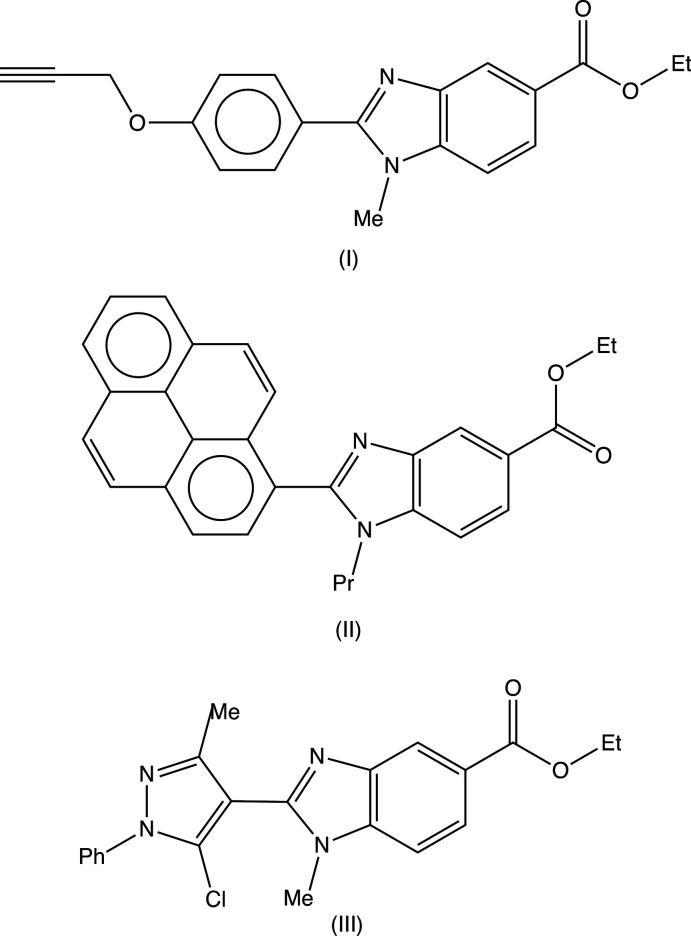


**Figure 4:**
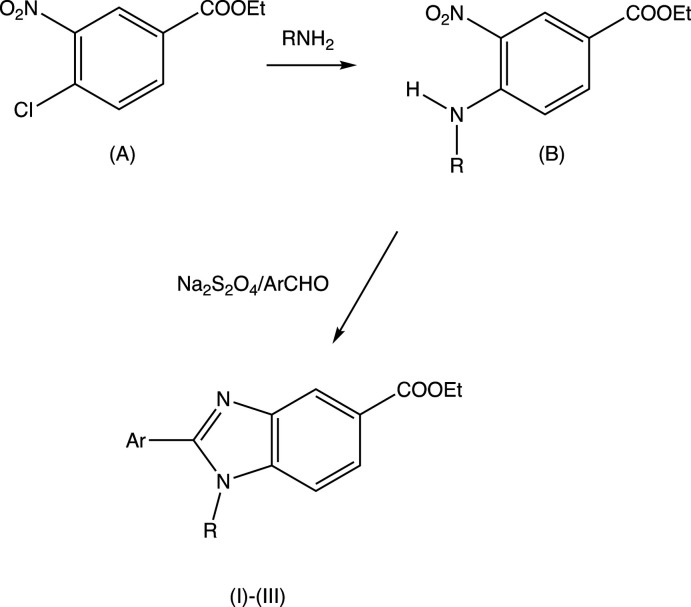
The synthetic pathway to compounds (I)–(III).

Compounds (I)–(III) were prepared from the commercially available precursor ethyl 4-chloro-3-nitro­benzoate (A) (Fig. 4 ▸), which readily undergoes nucleophilic substitution with primary amines to give the inter­mediates (B): subsequent reaction of (B) with sodium di­thio­nite in the presence of the appropriate aldehyde leads to the products (I)–(III) in overall yields of 58-68%.

## Structural commentary   

The mol­ecules of compounds (I)–(III) all exhibit disorder. In compound (I) (Fig. 1 ▸), the (prop-2-yn-1-oxy)phenyl unit is disordered over two sets of atomic sites having essentially equal occupancies, 0.506 (5) and 0.494 (5), such that the two orientations of the phenyl ring make almost identical dihedral angles with the adjacent imidazole ring, 27.8 (4) and 27.0 (4)° respectively, and with a dihedral angle of 54.7 (3)° between the planes of the two disorder components. The propyl group in compound (II) (Fig. 2 ▸) is disordered over two sets of atomic sites having occupancies 0.601 (8) and 0.399 (8), while in compound (III) (Fig. 3 ▸), the whole ester unit is disordered over two sets of atomic sites having occupancies 0.645 (7) and 0.355 (7); so, far from there being a single site for the methyl C atom in the ester unit (Manju *et al.*, 2018 ▸), there are two such sites in the present disorder model, separated by 0.931 (11) Å.

Despite the fact that atom O51 acts as a hydrogen-bond acceptor in both (II) and (III), although not in (I), the conformation of the ester unit in (II) is different from that in (I) and (III) (Figs. 1 ▸–4 ▸ ▸ ▸): the cause of this is unclear. The bond lengths in the pyrene fragment of compound (II) present some inter­esting features. While the distances in the rings containing atoms C22 and C27 are all typical of those in delocalized aromatic rings, those in the other two rings exhibit significant bond fixation (Glidewell & Lloyd, 1984 ▸). Thus the distances C24—C25 and C29—C210, 1.320 (3) and 1.342 (3) Å, are typical of double bonds (Allen *et al.*, 1987 ▸), while those for the bonds C23*A*—C24, C23*B*—C25*B*, C25—C25*A*, C28*A*—C29 and C210—C20*A* are all closely grouped in the *d* range 1.424 (3)–1.436 (3) Å, typical of single bonds carrying alkenyl or aromatic substituents (Allen *et al.*, 1987 ▸). Hence there can be no continuous peripheral delocalization in this unit.

## Supra­molecular features   

**Figure 5:**
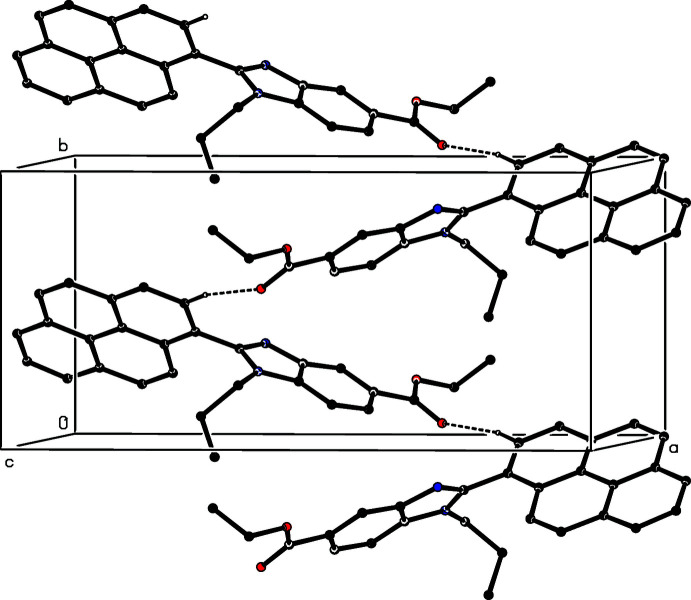
Part of the crystal structure of compound (II) showing the formation of a hydrogen-bonded *C*(10) chain parallel to [010]. Hydrogen bonds are drawn as dashed lines and, for the sake of clarity, the H atoms not involved in the motif shown have been omitted.

**Figure 6:**
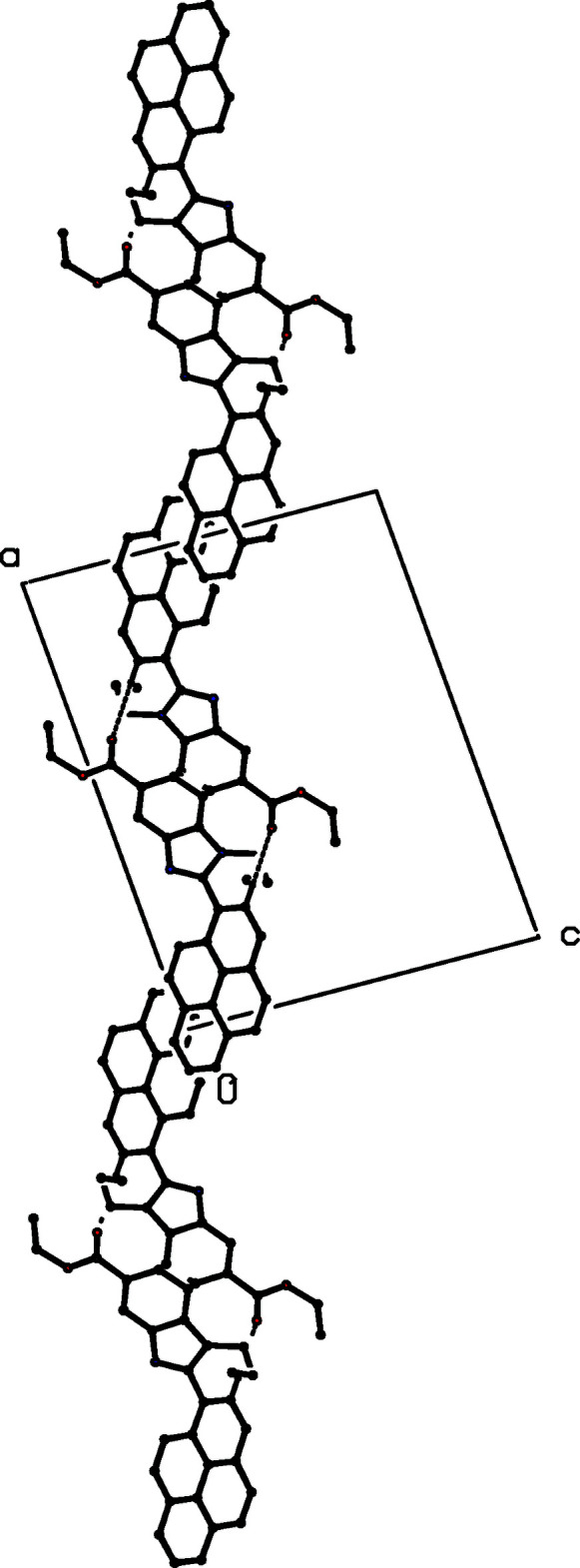
A projection along [010] of part of the crystal structure of compound (II) showing the formation of a π-stacked sheet of hydrogen-bonded chains. Hydrogen bonds are drawn as dashed lines and, for the sake of clarity, the H atoms not involved in the hydrogen bonding have been omitted.

The supra­molecular assembly in compounds (II) and (III) is very simple, but that in compound (I) is less straightforward. In compound (II), mol­ecules that are related by the 2_1_ screw axis along (0.5, *y*, 0.25) are linked by a C—H⋯O hydrogen bond (Table 1 ▸) to form a *C*(10) chain (Etter, 1990 ▸; Etter *et al.*, 1990 ▸; Bernstein *et al.*, 1995 ▸) running parallel to the [010] direction (Fig. 5 ▸). Two chains of this type, related to one another by inversion, pass through each unit cell, and these chains are linked by a π–π inter­action involving the terminal aromatic ring, containing atom C27 (Fig. 2 ▸). The terminal aromatic rings in the mol­ecules at (*x*, *y*, *z*) and (2 − *x*, 2 − *y*, 1 − *z*) are parallel with an inter­planar spacing of 3.430 (2) Å: the ring-centroid separation is 3.727 (2) Å and the ring-centroid offset is 1.459 (2) Å. This inter­action links the hydrogen-bonded chain around the screw axis along (0.5, *y*, 0.25) (Fig. 5 ▸) with the corresponding chains along (1.5, *y*, 0.75) and (−0.5, *y*, −0.25), hence generating a π-stacked sheet of hydrogen-bonded chains lying parallel to (10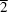) (Fig. 6 ▸). There is also another short C-H⋯O contact in the structure of (II), involving atom C11 (Table 1 ▸), but the C—H⋯O angle is very small, such that the inter­action energy here is likely to be negligibly small (Wood *et al.*, 2009 ▸). Hence, it is probably better to regard this as an adventitious contact rather than as a structurally significant inter­action: in any event, this contact would not influence the dimensionality of the supra­molecular assembly.

**Figure 7:**
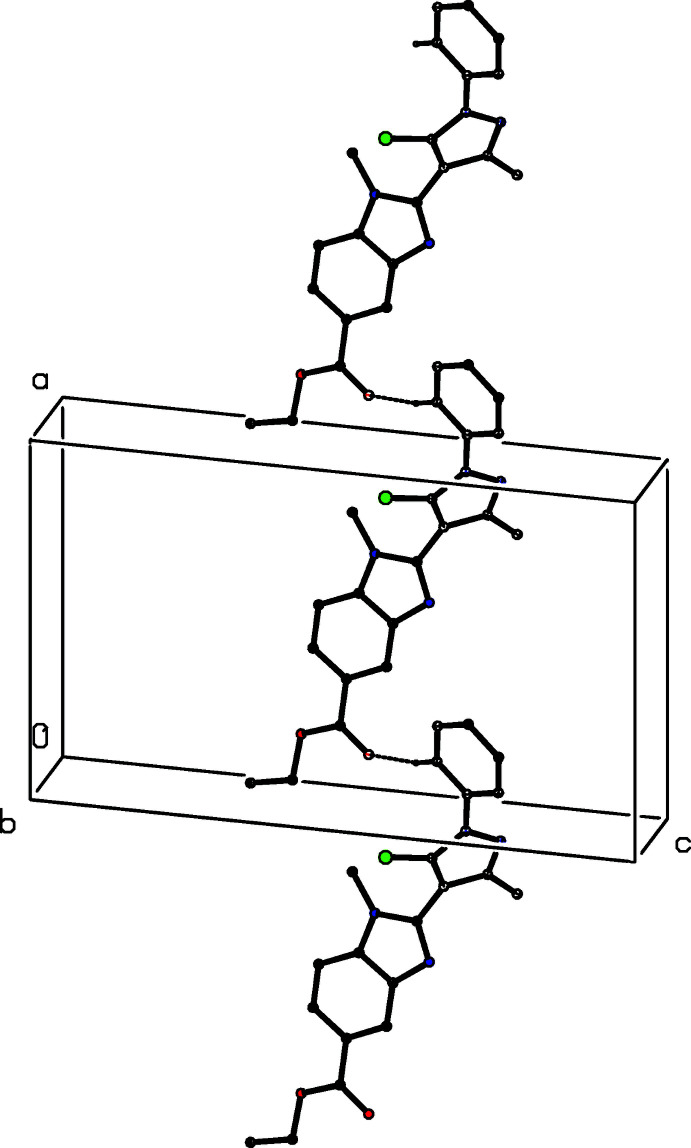
Part of the crystal structure of compound (III) showing the formation of a hydrogen-bonded *C*(13) chain parallel to [100]. Hydrogen bonds are drawn as dashed lines and, for the sake of clarity, the H atoms not involved in the motif shown have been omitted.

There is just one C—H⋯O hydrogen bond in the structure of compound (III), and its dimensions for the two disorder components are fairly similar, although the distances in the minor component are rather longer than those for the major form (Table 1 ▸); only the major disorder form needs to be considered. The hydrogen bond links mol­ecules that are related by translation to form a *C*(13) chain running parallel to the [100] direction (Fig. 7 ▸).

**Figure 8:**
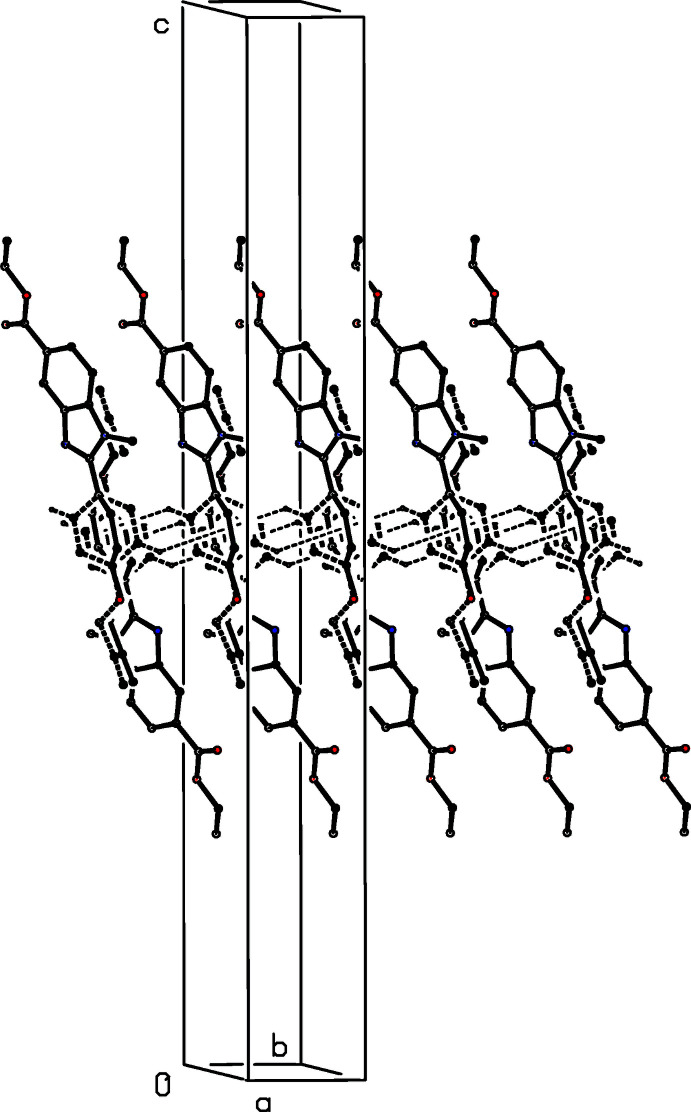
Part of the crystal structure of compound (I) showing the idealized ribbon along [010] that would result if all the hydrogen-bond donors and acceptors had unit occupancy. The disorder components are drawn using full and broken lines. Hydrogen bonds are drawn as dashed lines and, for the sake of clarity, the H atoms not involved in the motif shown have been omitted.

**Figure 9:**
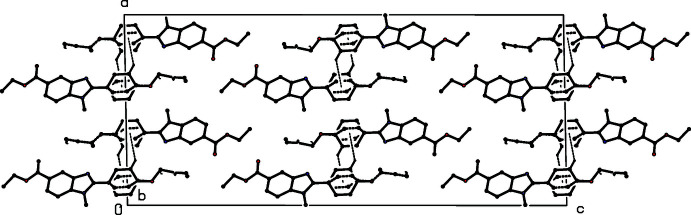
A projection along [010] of part of the crystal structure of compound (I) showing the arrangement of the punctuated ribbons within the unit cell. Hydrogen bonds are drawn as dashed lines and, for the sake of clarity, the H atoms not involved in the motifs shown have been omitted.

The structure of compound (I) contains three C—H⋯π(arene) hydrogen bonds, all involving the unfused aryl ring (Table 1 ▸), but the alkyne unit acts as neither donor nor acceptor. If all of the donors and acceptors were present with full occupancy, the effect of the hydrogen bonds would be to link the mol­ecules of (I) into a complex ribbon running parallel to the [010] direction (Fig. 8 ▸). However, in each of these hydrogen bonds, the donor and the acceptor form parts of different disorder components, so that the ribbon cannot be continuous, but it is punctuated into a succession of short fragments. The punctuated ribbon containing the reference mol­ecule lies along (0.25, *y*, 0.5) and there are symmetry-related ribbons along (0.25, *y*, 0), (0.75, *y*, 0) and (0.75, *y*, 0.5) (Fig. 9 ▸), but with no direction-specific inter­actions between adjacent ribbons.

## Database survey   

A representative example of a simple 2-substituted benzim­id­azole is provided by 2-(1-naphthyl­meth­yl)-1*H*-benzo[*d*]imidazole (IV) (Ding *et al.*, 2007 ▸); here the mol­ecules are linked by a single N—H⋯N hydrogen bond to form *C*(4) chains, which are themselves linked into sheets by a C—H⋯π(arene) hydrogen bond. Structures have been reported for a number of esters derived from substituted benzimidazole-5-carb­oxy­lic acids, including: ethyl 1-[3-(1*H*-imidazol-1-yl)prop­yl]-2-(4-chloro­phen­yl)-1*H*-benzo[*d*]imidazole- 5-caboxylate dehydrate (V) (Yoon *et al.*, 2011 ▸), where a combination of O—H⋯O and O—H⋯N hydrogen bonds generates complex sheets, rather than the three-dimensional assembly specified in the original report (Yoon *et al.*, 2011 ▸); the two closely related esters methyl 2-(4-bromo­phen­yl)-1-(5-*tert*-butyl-1*H*-pyrazol-3-yl)-1*H*-benzimidazole-5-carboxyl­ate (VI) (Cortés *et al.*, 2011 ▸) and octyl 1-(5-*tert*-butyl-1*H*-pyrazol-3-yl)-2-(4-chloro­phen­yl)1*H*-benzimidazole-5-carboxyl­ate (VII) (Cortés *et al.*, 2014 ▸), where the mol­ecules are linked into chains of edge-fused rings in (VI) by a combination of N—H⋯O and C—H⋯π(arene) hydrogen bonds, but into complex sheets in (VII) generated by a combination of N—H⋯N, C—H⋯N and C—H⋯O hydrogen bonds; and ethyl 1-(4-fluoro­benz­yl)-2-(4-meth­oxy­phen­yl)-1*H*-benzo[*d*]imidazole-5-carboxyl­ate (VIII) Naveen *et al.*, 2016 ▸), in which inversion-related pairs of mol­ecules are linked by C—H⋯O hydrogen bonds to form cyclic, centrosymmetric 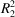
(22) dimers. It is notable that, in marked contrast to the compounds (I)–(III) reported here, none of compounds (IV)–(VIII) exhibits any disorder: we also note the contrasting patterns of supra­molecular inter­actions and assembly in the closely related esters (VI) and (VII).

## Synthesis and crystallization   

All reagents were obtained commercially, and all were used as received. For the synthesis of the inter­mediates of type (B) (Fig. 4 ▸), ethyl 4-chloro-3-nitro­benzoate (2.29 g, 0.01 mol) was dissolved in tetra­hydro­furan (20 ml) and 0.01 mol of the appropriate amine was added [0.80 ml of a 40% aqueous solution of methyl­amine when *R* = Me, or 0.059 g of propyl­amine when *R* = prop­yl], and these mixtures were then stirred at ambient temperature for 4 h. The resulting solid inter­mediates (B) were collected by filtration, dried in air and used without further purification. For the synthesis of the products (I)–(III), sodium di­thio­nite (1.74 g, 0.01 mol) was added to a mixture of (B) (0.01 mol) and the appropriate aldehyde (0.01 mol) [1.61 g of 4-propynyloxybenzaldehyde for (I); 2.30 g of pyrene-1-carboxaldehyde for (II); 2.20 g of 5-chloro-3-methyl-1-phenyl-1*H*-pyrazole-4-carboxaldehyde for (III)], in di­methyl­sulfoxide (30 ml). The reaction mixtures were then subjected to microwave irradiation (600 W) for 5 min for (I), 6.5 min for (II) and 6 min for (III). When the reactions were complete, as judged by thin-later chromatography, the resulting solid products were collected by filtration and dried in air.

Compound (I). Yield 62%, m.p. 465 K; IR (cm^−1^) 2218 (C≡C), 1708 (C=O), 1624(C=N); NMR (CDCl_3_) δ(^1^H) 1.37 (3H, *t*, *J* = 7.1 Hz, ester CH_3_), 3.48 (1H, *t*, *J* = 2.4 Hz, propynyl CH), 3.97 (3H, *s*, N—CH_3_), 4.36 (2H, *q*, *J* = 7.1 Hz, ester CH_2_), 4.90 (2H, *d*, *J* = 2.4 Hz, propynyl CH_2_), 7.22 (2H, *d*, *J* = 6.9 Hz) and 7.87 (2H, *d*, *J* = 6.9 Hz) (–C_6_H_4_–), 7.76 (1H, *d*, *J* = 8.6 Hz, H-7), 8.01 (1H, *dd*, *J* = 8.6 Hz and 1.3 Hz, H-6), 8.29 (1H, *d*, *J* = 1.3 Hz, H-4).

Compound (II). Yield 68%, m.p. 553 K; IR (cm^−1^) 1716 (C=O), 1615 (C=N); NMR (CDCl_3_) δ(^1^H) 0.62 (23H, *t*, *J* = 7.4 Hz, propyl CH_3_), 1.45 (3H, *t*, *J* = 7.1 Hz, ester CH_3_), 1.63 (2H, *m*, central CH_2_ of prop­yl), 4.06 (2H, *t*, *J* = 7.4 Hz), N—CH_2_), 4.45 (2H, *q*, *J* = 7.1 Hz, ester CH_2_), 7.53 (1H, *d*, *J* = 8.5 Hz, H-7), 7.91 (1H, *dd*, *J* = 8.5 Hz and 0.9 Hz, H-6), 8.05–8.32 (9H, *m*, pyrene), 8.67 (1H, *d*, *J* = 0.9 Hz, H-4).

Compound (III). Yield 58%, m.p. 435 K; IR (cm^−1^) 1703 (C=O), 1614 (C=N); NMR (CDCl_3_) δ(^1^H) 1.37 (3H, *t*, *J* = 7.0 Hz, ester CH_3_), 2.33 (3H, *s*, pyrazole CH_3_), 3.85 (3H, *s*, N—CH_3_), 4.36 (2H, *q*, *J* = 7.0 Hz, ester CH_2_), 7.51–8.30 (8H, *m*, aromatic).

Crystals suitable for single-crystal X-ray diffraction were grown by slow evaporation, at ambient temperature and in the presence of air, of solutions in ethanol-aceto­nitrile (initial composition 3:1 *v*/*v*).

## Refinement   

Crystal data, data collection and structure refinement details are summarized in Table 2 ▸. Two bad outlier reflections (2 2 9) and (1 1 19) were omitted from the final refinement of compound (I). All H atoms, apart from those in the minor disorder components, were located in difference maps. The H atoms were then all treated as riding atoms in geometrically idealized positions, with C—H distances of 0.93 Å (alkenyl, alkynyl and aromatic), 0.96 Å (CH_3_) or 0.97 Å (CH_2_), and with *U*_iso_(H) = *kU*_eq_(C), where *k* = 1.5 for the methyl groups, which were allowed to rotate but not to tilt, and 1.2 for all other H atoms. For the disorder components, the corresponding distances between bonding components and the 1,3 distances between non-bonding components were restrained to be equal, subject to s.u. values of 0.01 and 0.02 Å, respectively. In addition, for compound (I), similarity restraints were applied to the atoms of each orientation of the disordered aryl ring, while the anisotropic displacement parameters for corresponding pairs of atoms in the propyn­yloxy unit were constrained to be the same. Similarity restraints were applied to the displacement parameters of the terminal C atoms of the two disorder components of the propyl group in compound (II), and to those of corresponding pairs of atoms in the disordered ester unit of compound (III). Subject to these conditions, the site occupancies for the disordered fragments refined to 0.506 (5) and 0.494 (5) in (I), 0.601 (8) and 0.399 (8) in (II), and 0.645 (7) and 0.355 (7) in (III).

## Supplementary Material

Crystal structure: contains datablock(s) global, I, II, III. DOI: 10.1107/S2056989021003364/wm5604sup1.cif

Structure factors: contains datablock(s) I. DOI: 10.1107/S2056989021003364/wm5604Isup2.hkl

Structure factors: contains datablock(s) II. DOI: 10.1107/S2056989021003364/wm5604IIsup3.hkl

Structure factors: contains datablock(s) III. DOI: 10.1107/S2056989021003364/wm5604IIIsup4.hkl

Supporting information file. DOI: 10.1107/S2056989021003364/wm5604Isup5.cml

Supporting information file. DOI: 10.1107/S2056989021003364/wm5604IIsup6.cml

Supporting information file. DOI: 10.1107/S2056989021003364/wm5604IIIsup7.cml

CCDC references: 2074101, 2074100, 2074099

Additional supporting information:  crystallographic information; 3D view; checkCIF report

## Figures and Tables

**Table 1:** Hydrogen bonds and short inter­molecular contacts (Å, °) *Cg*1 and *Cg*2 represent the centroids of the rings (C31–C36) and (C21–C26), respectively.

Compound	*D*—H⋯*A*	*D*—H	H⋯*A*	*D*⋯*A*	*D*—H⋯*A*
(I)	C23—H23⋯*Cg*1^i^	0.93	2.77	3.412 (9)	127
	C26—H26⋯*Cg*1^ii^	0.93	2.87	3.508 (9)	127
	C35—H35⋯*Cg*2^iii^	0.93	2.87	3.555 (9)	131
(II)	C11—H11*A*⋯O51^iv^	0.97	2.51	3.253 (3)	133
	C22—H22⋯O51^iv^	0.93	2.37	3.290 (3)	168
(III)	C212—H212⋯O51^v^	0.93	2.54	3.460 (14)	168
	C212—H212⋯O61^v^	0.93	2.67	3.59 (2)	170

Symmetry codes: (i) *x*, 1 + *y*, *z*; (ii) *x*, −1 + *y*, *z*; (iii) {1\over 2} − *x*, {5\over 2} − *y*, 1 − *z*; (iv) 1 − *x*, {1\over 2} + *y*, {1\over 2} − *z*; (v) 1 + *x*, *y*, *z*.

**Table 2:** Experimental details

	(I)	(II)	(III)
Crystal data			
Chemical formula	C_20_H_18_N_2_O_3_	C_29_H_24_N_2_O_2_	C_21_H_19_ClN_4_O_2_
*M*_r_	334.36	432.50	394.85
Crystal system, space group	Monoclinic, *C*2/*c*	Monoclinic, *P*2_1_/*c*	Monoclinic, *P*2_1_/*n*
Temperature (K)	293	296	296
*a*, *b*, *c* (Å)	17.947 (2), 4.5907 (5), 41.305 (4)	18.467 (2), 8.6860 (8), 14.242 (1)	11.1095 (4), 9.5126 (4), 18.6747 (8)
β (°)	91.016 (8)	95.359 (7)	95.079 (4)
*V* (Å^3^)	3402.6 (6)	2274.5 (4)	1965.80 (14)
*Z*	8	4	4
Radiation type	Mo *K*α	Mo *K*α	Mo *K*α
μ (mm^−1^)	0.09	0.08	0.22
Crystal size (mm)	0.36 × 0.18 × 0.18	0.48 × 0.32 × 0.24	0.48 × 0.40 × 0.40

Data collection			
Diffractometer	Oxford Diffraction Xcalibur with Sapphire CCD	Oxford Diffraction Xcalibur with Sapphire CCD	Oxford Diffraction Xcalibur with Sapphire CCD
Absorption correction	Multi-scan (*CrysAlis RED*; Oxford Diffraction, 2009 ▸)	Multi-scan (*CrysAlis RED*; Oxford Diffraction, 2009 ▸)	Multi-scan (*CrysAlis RED*; Oxford Diffraction, 2009 ▸)
*T*_min_, *T*_max_	0.956, 0.984	0.905, 0.981	0.808, 0.916
No. of measured, independent and observed [*I* > 2σ(*I*)] reflections	7151, 3350, 2002	9384, 4691, 2593	8067, 4250, 3323
*R*_int_	0.038	0.022	0.013
(sin θ/λ)_max_ (Å^−1^)	0.618	0.629	0.656

Refinement			
*R*[*F* ^2^ > 2σ(*F* ^2^)], *wR*(*F* ^2^), *S*	0.068, 0.187, 1.04	0.052, 0.147, 1.01	0.040, 0.109, 1.02
No. of reflections	3350	4691	4250
No. of parameters	286	320	294
No. of restraints	93	9	28
H-atom treatment	H-atom parameters constrained	H-atom parameters constrained	H-atom parameters constrained
Δρ_max_, Δρ_min_ (e Å^−3^)	0.24, −0.23	0.15, −0.17	0.32, −0.29

Computer programs: *CrysAlis CCD* and *CrysAlis RED* (Oxford Diffraction, 2009 ▸), *SHELXT* (Sheldrick, 2015*a* ▸), *SHELXL2014* (Sheldrick, 2015*b* ▸) and *PLATON* (Spek, 2020 ▸).

## supplementary crystallographic information

### Ethyl 1-methyl-2-[4-(prop-2-yn-1-oxy)phenyl)-1H-benzimidazole-5-carboxylate (I) Crystal data

**Table:**

C_20_H_18_N_2_O_3_	*F*(000) = 1408
*M**_r_* = 334.36	*D*_x_ = 1.305 Mg m^−^^3^
Monoclinic, *C*2/*c*	Mo *K*α radiation, λ = 0.71073 Å
*a* = 17.947 (2) Å	Cell parameters from 3698 reflections
*b* = 4.5907 (5) Å	θ = 2.5–27.9°
*c* = 41.305 (4) Å	µ = 0.09 mm^−^^1^
β = 91.016 (8)°	*T* = 293 K
*V* = 3402.6 (6) Å^3^	Needle, yellow
*Z* = 8	0.36 × 0.18 × 0.18 mm

### Ethyl 1-methyl-2-[4-(prop-2-yn-1-oxy)phenyl)-1H-benzimidazole-5-carboxylate (I) Data collection

**Table:**

Oxford Diffraction Xcalibur with Sapphire CCD diffractometer	3350 independent reflections
Radiation source: Enhance (Mo) X-ray Source	2002 reflections with *I* > 2σ(*I*)
Graphite monochromator	*R*_int_ = 0.038
ω scans	θ_max_ = 26.0°, θ_min_ = 2.5°
Absorption correction: multi-scan (CrysAlis RED; Oxford Diffraction, 2009)	*h* = −20→22
*T*_min_ = 0.956, *T*_max_ = 0.984	*k* = −5→5
7151 measured reflections	*l* = −51→50

### Ethyl 1-methyl-2-[4-(prop-2-yn-1-oxy)phenyl)-1H-benzimidazole-5-carboxylate (I) Refinement

**Table:**

Refinement on *F*^2^	Primary atom site location: difference Fourier map
Least-squares matrix: full	Hydrogen site location: inferred from neighbouring sites
*R*[*F*^2^ > 2σ(*F*^2^)] = 0.068	H-atom parameters constrained
*wR*(*F*^2^) = 0.187	*w* = 1/[σ^2^(*F*_o_^2^) + (0.0762*P*)^2^ + 3.8366*P*] where *P* = (*F*_o_^2^ + 2*F*_c_^2^)/3
*S* = 1.04	(Δ/σ)_max_ < 0.001
3350 reflections	Δρ_max_ = 0.24 e Å^−^^3^
286 parameters	Δρ_min_ = −0.23 e Å^−^^3^
93 restraints	

### Ethyl 1-methyl-2-[4-(prop-2-yn-1-oxy)phenyl)-1H-benzimidazole-5-carboxylate (I) Special details

**Table:**

Geometry. All esds (except the esd in the dihedral angle between two l.s. planes) are estimated using the full covariance matrix. The cell esds are taken into account individually in the estimation of esds in distances, angles and torsion angles; correlations between esds in cell parameters are only used when they are defined by crystal symmetry. An approximate (isotropic) treatment of cell esds is used for estimating esds involving l.s. planes.

### Ethyl 1-methyl-2-[4-(prop-2-yn-1-oxy)phenyl)-1H-benzimidazole-5-carboxylate (I) Fractional atomic coordinates and isotropic or equivalent isotropic displacement parameters (Å^2^)

**Table:**

	*x*	*y*	*z*	*U*_iso_*/*U*_eq_	Occ. (<1)
N1	0.43626 (12)	1.0912 (5)	0.59851 (6)	0.0489 (6)	
C2	0.38294 (15)	0.9896 (6)	0.57634 (7)	0.0446 (7)	
N3	0.33476 (13)	0.8107 (6)	0.58926 (6)	0.0512 (7)	
C3A	0.35648 (15)	0.7906 (7)	0.62171 (7)	0.0495 (7)	
C4	0.32625 (17)	0.6286 (8)	0.64653 (7)	0.0566 (8)	
H4	0.2840	0.5148	0.6429	0.068*	
C5	0.36027 (17)	0.6401 (7)	0.67670 (7)	0.0544 (8)	
C6	0.42355 (19)	0.8131 (8)	0.68213 (8)	0.0637 (9)	
H6	0.4455	0.8170	0.7027	0.076*	
C7	0.45376 (18)	0.9757 (8)	0.65814 (8)	0.0619 (9)	
H7	0.4957	1.0908	0.6620	0.074*	
C7A	0.41952 (16)	0.9633 (7)	0.62761 (7)	0.0482 (7)	
C11	0.4975 (2)	1.2924 (9)	0.59433 (9)	0.0821 (12)	
H11A	0.5175	1.2689	0.5731	0.123*	
H11B	0.5358	1.2531	0.6103	0.123*	
H11C	0.4800	1.4885	0.5969	0.123*	
C21	0.38139 (14)	1.0664 (6)	0.54171 (7)	0.0411 (6)	0.494 (5)
C22	0.4071 (3)	1.3274 (12)	0.52923 (13)	0.0475 (16)	0.494 (5)
H22	0.4282	1.4637	0.5433	0.057*	0.494 (5)
C23	0.4023 (3)	1.3911 (13)	0.49670 (13)	0.0467 (15)	0.494 (5)
H23	0.4203	1.5675	0.4890	0.056*	0.494 (5)
C24	0.370 (2)	1.190 (10)	0.4754 (3)	0.043 (4)	0.494 (5)
C25	0.3444 (3)	0.9279 (13)	0.48742 (13)	0.0438 (15)	0.494 (5)
H25	0.3234	0.7913	0.4734	0.053*	0.494 (5)
C26	0.3497 (3)	0.8692 (12)	0.52000 (12)	0.0433 (15)	0.494 (5)
H26	0.3316	0.6930	0.5277	0.052*	0.494 (5)
O24	0.374 (4)	1.250 (18)	0.4431 (5)	0.059 (3)	0.494 (5)
C27	0.3276 (10)	1.101 (4)	0.4211 (3)	0.064 (4)	0.494 (5)
H27A	0.2762	1.1196	0.4276	0.076*	0.494 (5)
H27B	0.3404	0.8960	0.4209	0.076*	0.494 (5)
C28	0.336 (2)	1.224 (9)	0.3893 (4)	0.066 (7)	0.494 (5)
C29	0.347 (2)	1.295 (7)	0.3629 (5)	0.087 (6)	0.494 (5)
H29	0.3550	1.3533	0.3417	0.104*	0.494 (5)
C31	0.38139 (14)	1.0664 (6)	0.54171 (7)	0.0411 (6)	0.506 (5)
C32	0.4451 (3)	1.1350 (13)	0.52509 (12)	0.0436 (14)	0.506 (5)
H32	0.4907	1.1427	0.5361	0.052*	0.506 (5)
C33	0.4418 (3)	1.1920 (13)	0.49242 (12)	0.0432 (15)	0.506 (5)
H33	0.4850	1.2396	0.4815	0.052*	0.506 (5)
C34	0.3741 (18)	1.179 (9)	0.4755 (3)	0.041 (3)	0.506 (5)
C35	0.3100 (3)	1.1029 (14)	0.49176 (12)	0.0417 (14)	0.506 (5)
H35	0.2646	1.0900	0.4806	0.050*	0.506 (5)
C36	0.3141 (3)	1.0467 (14)	0.52448 (12)	0.0451 (15)	0.506 (5)
H36	0.2711	0.9945	0.5353	0.054*	0.506 (5)
O34	0.373 (4)	1.268 (18)	0.4439 (5)	0.059 (3)	0.506 (5)
C37	0.3125 (9)	1.196 (4)	0.4235 (3)	0.064 (4)	0.506 (5)
H37A	0.2675	1.2874	0.4313	0.076*	0.506 (5)
H37B	0.3051	0.9866	0.4235	0.076*	0.506 (5)
C38	0.328 (2)	1.296 (9)	0.3913 (3)	0.066 (7)	0.506 (5)
C39	0.331 (2)	1.388 (6)	0.3653 (5)	0.087 (6)	0.506 (5)
H39	0.3329	1.4621	0.3443	0.104*	0.506 (5)
C51	0.3279 (2)	0.4646 (8)	0.70302 (8)	0.0633 (9)	
O51	0.26896 (15)	0.3386 (7)	0.70080 (5)	0.0852 (9)	
O52	0.37052 (14)	0.4589 (6)	0.72963 (5)	0.0762 (8)	
C52	0.3433 (2)	0.2891 (10)	0.75689 (8)	0.0840 (12)	
H52A	0.2991	0.3798	0.7655	0.101*	
H52B	0.3304	0.0939	0.7498	0.101*	
C53	0.4009 (3)	0.2764 (15)	0.78145 (10)	0.125 (2)	
H53A	0.3820	0.1822	0.8004	0.187*	
H53B	0.4166	0.4704	0.7869	0.187*	
H53C	0.4425	0.1681	0.7735	0.187*	

### Ethyl 1-methyl-2-[4-(prop-2-yn-1-oxy)phenyl)-1H-benzimidazole-5-carboxylate (I) Atomic displacement parameters (Å^2^)

**Table:**

	*U*^11^	*U*^22^	*U*^33^	*U*^12^	*U*^13^	*U*^23^
N1	0.0422 (13)	0.0431 (14)	0.0613 (16)	−0.0045 (11)	−0.0053 (11)	−0.0034 (12)
C2	0.0377 (14)	0.0422 (17)	0.0538 (17)	0.0026 (13)	−0.0032 (12)	−0.0030 (14)
N3	0.0463 (13)	0.0586 (16)	0.0486 (14)	−0.0093 (13)	−0.0047 (11)	−0.0011 (12)
C3A	0.0455 (16)	0.0568 (19)	0.0459 (16)	0.0009 (15)	−0.0051 (13)	−0.0032 (15)
C4	0.0532 (17)	0.065 (2)	0.0511 (18)	−0.0082 (16)	−0.0030 (14)	−0.0004 (16)
C5	0.0539 (18)	0.061 (2)	0.0477 (18)	0.0026 (16)	−0.0044 (14)	−0.0045 (16)
C6	0.065 (2)	0.072 (2)	0.0534 (19)	−0.0010 (18)	−0.0143 (16)	−0.0040 (18)
C7	0.0579 (19)	0.067 (2)	0.060 (2)	−0.0075 (17)	−0.0166 (16)	−0.0065 (18)
C7A	0.0456 (16)	0.0505 (18)	0.0483 (17)	0.0038 (14)	−0.0041 (13)	−0.0057 (15)
C11	0.077 (2)	0.087 (3)	0.081 (3)	−0.040 (2)	−0.018 (2)	0.005 (2)
C21	0.0351 (13)	0.0366 (15)	0.0517 (16)	0.0024 (12)	−0.0005 (12)	−0.0031 (13)
C22	0.045 (3)	0.041 (3)	0.057 (3)	−0.001 (3)	−0.007 (3)	−0.008 (3)
C23	0.041 (3)	0.039 (3)	0.060 (3)	−0.006 (3)	−0.003 (3)	0.004 (3)
C24	0.037 (6)	0.038 (6)	0.055 (6)	−0.001 (6)	−0.003 (5)	−0.002 (5)
C25	0.037 (3)	0.040 (3)	0.055 (3)	−0.004 (3)	−0.005 (2)	−0.004 (3)
C26	0.034 (3)	0.038 (3)	0.058 (3)	−0.003 (3)	−0.001 (2)	0.002 (3)
O24	0.0579 (19)	0.069 (7)	0.0488 (14)	−0.017 (4)	−0.0034 (16)	0.001 (3)
C27	0.052 (6)	0.079 (10)	0.059 (3)	−0.019 (6)	−0.006 (3)	0.001 (4)
C28	0.062 (8)	0.078 (17)	0.057 (2)	−0.015 (10)	−0.004 (3)	−0.010 (5)
C29	0.099 (14)	0.104 (17)	0.057 (3)	−0.015 (10)	0.000 (4)	0.001 (7)
C31	0.0351 (13)	0.0366 (15)	0.0517 (16)	0.0024 (12)	−0.0005 (12)	−0.0031 (13)
C32	0.033 (3)	0.042 (3)	0.056 (3)	0.002 (2)	−0.005 (2)	−0.007 (3)
C33	0.034 (3)	0.042 (3)	0.053 (3)	−0.003 (3)	0.008 (2)	−0.002 (3)
C34	0.037 (6)	0.041 (6)	0.046 (5)	0.001 (5)	0.004 (5)	−0.002 (5)
C35	0.035 (3)	0.042 (3)	0.048 (3)	0.000 (3)	−0.006 (2)	0.002 (3)
C36	0.036 (3)	0.047 (3)	0.053 (3)	−0.005 (3)	0.005 (2)	0.000 (3)
O34	0.0579 (19)	0.069 (7)	0.0488 (14)	−0.017 (4)	−0.0034 (16)	0.001 (3)
C37	0.052 (6)	0.079 (10)	0.059 (3)	−0.019 (6)	−0.006 (3)	0.001 (4)
C38	0.062 (8)	0.078 (17)	0.057 (2)	−0.015 (10)	−0.004 (3)	−0.010 (5)
C39	0.099 (14)	0.104 (17)	0.057 (3)	−0.015 (10)	0.000 (4)	0.001 (7)
C51	0.066 (2)	0.075 (2)	0.0492 (19)	0.0015 (19)	−0.0055 (16)	−0.0023 (18)
O51	0.0768 (17)	0.121 (2)	0.0578 (15)	−0.0251 (17)	−0.0062 (12)	0.0157 (15)
O52	0.0784 (16)	0.098 (2)	0.0520 (13)	−0.0068 (15)	−0.0122 (12)	0.0099 (13)
C52	0.094 (3)	0.103 (3)	0.055 (2)	−0.005 (2)	−0.0054 (19)	0.017 (2)
C53	0.118 (4)	0.180 (6)	0.075 (3)	−0.014 (4)	−0.024 (3)	0.039 (3)

### Ethyl 1-methyl-2-[4-(prop-2-yn-1-oxy)phenyl)-1H-benzimidazole-5-carboxylate (I) Geometric parameters (Å, º)

**Table:**

N1—C7A	1.376 (4)	O24—C27	1.403 (18)
N1—C2	1.393 (3)	C27—C28	1.439 (7)
N1—C11	1.449 (4)	C27—H27A	0.9700
C2—N3	1.313 (4)	C27—H27B	0.9700
C2—C21	1.473 (4)	C28—C29	1.156 (8)
N3—C3A	1.392 (4)	C29—H29	0.9300
C3A—C4	1.385 (4)	C32—C33	1.375 (6)
C3A—C7A	1.399 (4)	C32—H32	0.9300
C4—C5	1.379 (4)	C33—C34	1.39 (2)
C4—H4	0.9300	C33—H33	0.9300
C5—C6	1.401 (5)	C34—O34	1.369 (9)
C5—C51	1.480 (5)	C34—C35	1.39 (2)
C6—C7	1.361 (5)	C35—C36	1.376 (6)
C6—H6	0.9300	C35—H35	0.9300
C7—C7A	1.394 (4)	C36—H36	0.9300
C7—H7	0.9300	O34—C37	1.404 (18)
C11—H11A	0.9600	C37—C38	1.440 (7)
C11—H11B	0.9600	C37—H37A	0.9700
C11—H11C	0.9600	C37—H37B	0.9700
C21—C22	1.386 (6)	C38—C39	1.157 (8)
C21—C26	1.389 (5)	C39—H39	0.9300
C22—C23	1.376 (7)	C51—O51	1.208 (4)
C22—H22	0.9300	C51—O52	1.328 (4)
C23—C24	1.39 (2)	O52—C52	1.460 (4)
C23—H23	0.9300	C52—C53	1.436 (5)
C24—O24	1.368 (9)	C52—H52A	0.9700
C24—C25	1.38 (2)	C52—H52B	0.9700
C25—C26	1.374 (6)	C53—H53A	0.9600
C25—H25	0.9300	C53—H53B	0.9600
C26—H26	0.9300	C53—H53C	0.9600
			
C7A—N1—C2	105.9 (2)	C25—C26—H26	119.2
C7A—N1—C11	123.7 (3)	C21—C26—H26	119.2
C2—N1—C11	130.5 (3)	C24—O24—C27	119.6 (12)
N3—C2—N1	113.1 (2)	O24—C27—C28	109.0 (11)
N3—C2—C21	122.9 (2)	O24—C27—H27A	109.9
N1—C2—C21	124.0 (3)	C28—C27—H27A	109.9
C2—N3—C3A	104.9 (2)	O24—C27—H27B	109.9
C4—C3A—N3	129.9 (3)	C28—C27—H27B	109.9
C4—C3A—C7A	120.1 (3)	H27A—C27—H27B	108.3
N3—C3A—C7A	110.0 (3)	C29—C28—C27	173 (5)
C5—C4—C3A	118.4 (3)	C28—C29—H29	180.0
C5—C4—H4	120.8	C33—C32—H32	119.7
C3A—C4—H4	120.8	C32—C33—C34	120.4 (7)
C4—C5—C6	120.8 (3)	C32—C33—H33	119.8
C4—C5—C51	118.0 (3)	C34—C33—H33	119.8
C6—C5—C51	121.2 (3)	O34—C34—C35	123 (3)
C7—C6—C5	121.8 (3)	O34—C34—C33	117 (2)
C7—C6—H6	119.1	C35—C34—C33	119.6 (5)
C5—C6—H6	119.1	C36—C35—C34	119.4 (7)
C6—C7—C7A	117.5 (3)	C36—C35—H35	120.3
C6—C7—H7	121.3	C34—C35—H35	120.3
C7A—C7—H7	121.3	C35—C36—H36	119.3
N1—C7A—C7	132.3 (3)	C34—O34—C37	119.8 (12)
N1—C7A—C3A	106.1 (2)	O34—C37—C38	108.8 (10)
C7—C7A—C3A	121.5 (3)	O34—C37—H37A	109.9
N1—C11—H11A	109.5	C38—C37—H37A	109.9
N1—C11—H11B	109.5	O34—C37—H37B	109.9
H11A—C11—H11B	109.5	C38—C37—H37B	109.9
N1—C11—H11C	109.5	H37A—C37—H37B	108.3
H11A—C11—H11C	109.5	C39—C38—C37	171 (4)
H11B—C11—H11C	109.5	C38—C39—H39	180.0
C22—C21—C26	117.3 (4)	O51—C51—O52	123.0 (3)
C22—C21—C2	124.6 (3)	O51—C51—C5	124.0 (3)
C26—C21—C2	118.1 (3)	O52—C51—C5	113.0 (3)
C23—C22—C21	122.1 (5)	C51—O52—C52	116.9 (3)
C23—C22—H22	119.0	C53—C52—O52	108.7 (3)
C21—C22—H22	119.0	C53—C52—H52A	110.0
C22—C23—C24	119.5 (8)	O52—C52—H52A	110.0
C22—C23—H23	120.3	C53—C52—H52B	110.0
C24—C23—H23	120.3	O52—C52—H52B	110.0
O24—C24—C25	123 (3)	H52A—C52—H52B	108.3
O24—C24—C23	117 (2)	C52—C53—H53A	109.5
C25—C24—C23	119.3 (5)	C52—C53—H53B	109.5
C26—C25—C24	120.2 (8)	H53A—C53—H53B	109.5
C26—C25—H25	119.9	C52—C53—H53C	109.5
C24—C25—H25	119.9	H53A—C53—H53C	109.5
C25—C26—C21	121.6 (5)	H53B—C53—H53C	109.5
			
C7A—N1—C2—N3	0.1 (3)	N1—C2—C21—C26	152.4 (4)
C11—N1—C2—N3	−178.9 (3)	C26—C21—C22—C23	−0.5 (7)
C7A—N1—C2—C21	−178.2 (2)	C2—C21—C22—C23	−178.1 (4)
C11—N1—C2—C21	2.8 (5)	C21—C22—C23—C24	0 (3)
N1—C2—N3—C3A	0.0 (3)	C22—C23—C24—O24	−173 (5)
C21—C2—N3—C3A	178.4 (3)	C22—C23—C24—C25	0 (5)
C2—N3—C3A—C4	−178.9 (3)	O24—C24—C25—C26	173 (5)
C2—N3—C3A—C7A	−0.2 (3)	C23—C24—C25—C26	1 (5)
N3—C3A—C4—C5	177.9 (3)	C24—C25—C26—C21	−1 (3)
C7A—C3A—C4—C5	−0.7 (5)	C22—C21—C26—C25	0.5 (7)
C3A—C4—C5—C6	0.4 (5)	C2—C21—C26—C25	178.3 (4)
C3A—C4—C5—C51	−179.6 (3)	C25—C24—O24—C27	24 (11)
C4—C5—C6—C7	0.1 (5)	C23—C24—O24—C27	−163 (6)
C51—C5—C6—C7	−179.9 (3)	C24—O24—C27—C28	174 (7)
C5—C6—C7—C7A	−0.3 (5)	C32—C33—C34—O34	173 (5)
C2—N1—C7A—C7	178.1 (3)	C32—C33—C34—C35	−1 (5)
C11—N1—C7A—C7	−2.8 (5)	O34—C34—C35—C36	−173 (5)
C2—N1—C7A—C3A	−0.2 (3)	C33—C34—C35—C36	1 (5)
C11—N1—C7A—C3A	178.9 (3)	C35—C34—O34—C37	−21 (11)
C6—C7—C7A—N1	−178.2 (3)	C33—C34—O34—C37	165 (6)
C6—C7—C7A—C3A	0.0 (5)	C34—O34—C37—C38	−176 (7)
C4—C3A—C7A—N1	179.1 (3)	C4—C5—C51—O51	−8.8 (5)
N3—C3A—C7A—N1	0.3 (3)	C6—C5—C51—O51	171.1 (4)
C4—C3A—C7A—C7	0.5 (5)	C4—C5—C51—O52	171.1 (3)
N3—C3A—C7A—C7	−178.3 (3)	C6—C5—C51—O52	−8.9 (5)
N3—C2—C21—C22	151.8 (4)	O51—C51—O52—C52	0.3 (5)
N1—C2—C21—C22	−30.0 (5)	C5—C51—O52—C52	−179.6 (3)
N3—C2—C21—C26	−25.8 (5)	C51—O52—C52—C53	172.2 (4)

### Ethyl 1-methyl-2-[4-(prop-2-yn-1-oxy)phenyl)-1H-benzimidazole-5-carboxylate (I) Hydrogen-bond geometry (Å, º)

**Table:**

*D*—H···*A*	*D*—H	H···*A*	*D*···*A*	*D*—H···*A*
C23—H23···*Cg*1^i^	0.93	2.77	3.412 (9)	127
C26—H26···*Cg*1^ii^	0.93	2.87	3.508 (9)	127
C35—H35···*Cg*2^iii^	0.93	2.87	3.555 (9)	131

Symmetry codes: (i) *x*, *y*+1, *z*; (ii) *x*, *y*−1, *z*; (iii) −*x*+1/2, −*y*+5/2, −*z*+1.

### Ethyl 1-propyl-2-(pyren-1-yl)-1H-benzimidazole-5-carboxylate (II) Crystal data

**Table:**

C_29_H_24_N_2_O_2_	*F*(000) = 912
*M**_r_* = 432.50	*D*_x_ = 1.263 Mg m^−^^3^
Monoclinic, *P*2_1_/*c*	Mo *K*α radiation, λ = 0.71073 Å
*a* = 18.467 (2) Å	Cell parameters from 4909 reflections
*b* = 8.6860 (8) Å	θ = 2.6–27.8°
*c* = 14.242 (1) Å	µ = 0.08 mm^−^^1^
β = 95.359 (7)°	*T* = 296 K
*V* = 2274.5 (4) Å^3^	Block, yellow
*Z* = 4	0.48 × 0.32 × 0.24 mm

### Ethyl 1-propyl-2-(pyren-1-yl)-1H-benzimidazole-5-carboxylate (II) Data collection

**Table:**

Oxford Diffraction Xcalibur with Sapphire CCD diffractometer	4691 independent reflections
Radiation source: Enhance (Mo) X-ray Source	2593 reflections with *I* > 2σ(*I*)
Graphite monochromator	*R*_int_ = 0.022
ω scans	θ_max_ = 26.6°, θ_min_ = 2.6°
Absorption correction: multi-scan (CrysAlis RED; Oxford Diffraction, 2009)	*h* = −18→23
*T*_min_ = 0.905, *T*_max_ = 0.981	*k* = −10→7
9384 measured reflections	*l* = −14→17

### Ethyl 1-propyl-2-(pyren-1-yl)-1H-benzimidazole-5-carboxylate (II) Refinement

**Table:**

Refinement on *F*^2^	Primary atom site location: difference Fourier map
Least-squares matrix: full	Hydrogen site location: inferred from neighbouring sites
*R*[*F*^2^ > 2σ(*F*^2^)] = 0.052	H-atom parameters constrained
*wR*(*F*^2^) = 0.147	*w* = 1/[σ^2^(*F*_o_^2^) + (0.0634*P*)^2^ + 0.389*P*] where *P* = (*F*_o_^2^ + 2*F*_c_^2^)/3
*S* = 1.01	(Δ/σ)_max_ < 0.001
4691 reflections	Δρ_max_ = 0.15 e Å^−^^3^
320 parameters	Δρ_min_ = −0.17 e Å^−^^3^
9 restraints	

### Ethyl 1-propyl-2-(pyren-1-yl)-1H-benzimidazole-5-carboxylate (II) Special details

**Table:**

Geometry. All esds (except the esd in the dihedral angle between two l.s. planes) are estimated using the full covariance matrix. The cell esds are taken into account individually in the estimation of esds in distances, angles and torsion angles; correlations between esds in cell parameters are only used when they are defined by crystal symmetry. An approximate (isotropic) treatment of cell esds is used for estimating esds involving l.s. planes.

### Ethyl 1-propyl-2-(pyren-1-yl)-1H-benzimidazole-5-carboxylate (II) Fractional atomic coordinates and isotropic or equivalent isotropic displacement parameters (Å^2^)

**Table:**

	*x*	*y*	*z*	*U*_iso_*/*U*_eq_	Occ. (<1)
N1	0.65691 (9)	0.7408 (2)	0.23708 (12)	0.0594 (5)	
C2	0.69734 (11)	0.8183 (3)	0.30919 (15)	0.0568 (5)	
N3	0.66307 (9)	0.8333 (2)	0.38560 (12)	0.0614 (5)	
C3A	0.59665 (10)	0.7619 (2)	0.36420 (15)	0.0551 (5)	
C4	0.53959 (11)	0.7415 (2)	0.41957 (16)	0.0595 (6)	
H4	0.5418	0.7821	0.4802	0.071*	
C5	0.47943 (11)	0.6597 (3)	0.38271 (16)	0.0608 (6)	
C6	0.47627 (13)	0.6019 (3)	0.29037 (18)	0.0747 (7)	
H6	0.4351	0.5478	0.2667	0.090*	
C7	0.53147 (13)	0.6219 (3)	0.23378 (17)	0.0755 (7)	
H7	0.5285	0.5835	0.1726	0.091*	
C7A	0.59237 (11)	0.7029 (2)	0.27223 (15)	0.0581 (6)	
C21	0.77089 (11)	0.8787 (2)	0.29866 (14)	0.0543 (5)	
C22	0.78055 (13)	0.9853 (3)	0.22785 (15)	0.0660 (6)	
H22	0.7404	1.0178	0.1886	0.079*	
C23	0.84795 (13)	1.0433 (3)	0.21471 (16)	0.0686 (6)	
H23	0.8527	1.1143	0.1669	0.082*	
C23A	0.90911 (12)	0.9973 (2)	0.27184 (15)	0.0575 (5)	
C24	0.98059 (14)	1.0554 (3)	0.26061 (18)	0.0760 (7)	
H24	0.9867	1.1249	0.2123	0.091*	
C25	1.03788 (14)	1.0128 (3)	0.3170 (2)	0.0791 (7)	
H25	1.0832	1.0530	0.3070	0.095*	
C25A	1.03266 (11)	0.9067 (3)	0.39295 (16)	0.0623 (6)	
C26	1.09224 (13)	0.8616 (3)	0.4536 (2)	0.0786 (7)	
H26	1.1379	0.9020	0.4459	0.094*	
C27	1.08436 (14)	0.7582 (3)	0.5245 (2)	0.0823 (8)	
H27	1.1249	0.7296	0.5644	0.099*	
C28	1.01793 (13)	0.6961 (3)	0.53772 (17)	0.0752 (7)	
H28	1.0139	0.6253	0.5859	0.090*	
C28A	0.95602 (11)	0.7384 (3)	0.47925 (15)	0.0575 (5)	
C29	0.88535 (12)	0.6795 (3)	0.49081 (16)	0.0672 (6)	
H29	0.8800	0.6088	0.5387	0.081*	
C210	0.82629 (11)	0.7225 (3)	0.43487 (15)	0.0606 (6)	
H210	0.7812	0.6814	0.4452	0.073*	
C20A	0.83110 (10)	0.8306 (2)	0.35956 (13)	0.0488 (5)	
C23B	0.90095 (10)	0.8902 (2)	0.34558 (13)	0.0492 (5)	
C25B	0.96325 (10)	0.8449 (2)	0.40574 (14)	0.0516 (5)	
C11	0.67891 (14)	0.6952 (3)	0.14429 (16)	0.0754 (7)	0.601 (8)
H11A	0.6917	0.7873	0.1110	0.090*	0.601 (8)
H11B	0.6374	0.6486	0.1082	0.090*	0.601 (8)
C12	0.7423 (3)	0.5839 (5)	0.1472 (3)	0.0673 (16)	0.601 (8)
H12A	0.7850	0.6317	0.1796	0.081*	0.601 (8)
H12B	0.7529	0.5605	0.0833	0.081*	0.601 (8)
C13	0.7256 (9)	0.4347 (13)	0.1976 (10)	0.118 (5)	0.601 (8)
H13A	0.7673	0.3684	0.2005	0.177*	0.601 (8)
H13B	0.6850	0.3842	0.1636	0.177*	0.601 (8)
H13C	0.7139	0.4578	0.2604	0.177*	0.601 (8)
C14	0.67891 (14)	0.6952 (3)	0.14429 (16)	0.0754 (7)	0.399 (8)
H14A	0.7242	0.7458	0.1333	0.090*	0.399 (8)
H14B	0.6420	0.7269	0.0951	0.090*	0.399 (8)
C15	0.6886 (8)	0.5245 (9)	0.1412 (6)	0.125 (5)	0.399 (8)
H15A	0.7010	0.4947	0.0791	0.150*	0.399 (8)
H15B	0.6431	0.4746	0.1521	0.150*	0.399 (8)
C16	0.7483 (12)	0.4707 (19)	0.2155 (15)	0.114 (6)	0.399 (8)
H16A	0.7573	0.3630	0.2071	0.172*	0.399 (8)
H16B	0.7331	0.4878	0.2773	0.172*	0.399 (8)
H16C	0.7921	0.5277	0.2087	0.172*	0.399 (8)
C51	0.41793 (12)	0.6277 (3)	0.43984 (19)	0.0696 (6)	
O51	0.36732 (9)	0.5459 (2)	0.41475 (14)	0.1016 (6)	
O52	0.42445 (8)	0.6961 (2)	0.52331 (13)	0.0863 (5)	
C52	0.36844 (13)	0.6652 (4)	0.58661 (19)	0.0901 (8)	
H52A	0.3884	0.6817	0.6513	0.108*	
H52B	0.3535	0.5583	0.5803	0.108*	
C53	0.30450 (18)	0.7653 (4)	0.5657 (2)	0.1197 (12)	
H53A	0.2714	0.7513	0.6132	0.180*	
H53B	0.2806	0.7389	0.5051	0.180*	
H53C	0.3198	0.8709	0.5653	0.180*	

### Ethyl 1-propyl-2-(pyren-1-yl)-1H-benzimidazole-5-carboxylate (II) Atomic displacement parameters (Å^2^)

**Table:**

	*U*^11^	*U*^22^	*U*^33^	*U*^12^	*U*^13^	*U*^23^
N1	0.0625 (11)	0.0609 (11)	0.0535 (10)	0.0061 (9)	−0.0013 (8)	−0.0059 (9)
C2	0.0557 (12)	0.0581 (14)	0.0559 (13)	0.0049 (11)	0.0022 (10)	−0.0014 (10)
N3	0.0505 (10)	0.0701 (12)	0.0635 (11)	−0.0064 (9)	0.0052 (8)	−0.0140 (9)
C3A	0.0488 (11)	0.0558 (13)	0.0587 (12)	0.0018 (10)	−0.0052 (9)	−0.0074 (10)
C4	0.0533 (12)	0.0630 (14)	0.0607 (13)	−0.0022 (11)	−0.0032 (10)	−0.0086 (11)
C5	0.0504 (12)	0.0593 (14)	0.0700 (14)	−0.0004 (10)	−0.0096 (10)	0.0005 (11)
C6	0.0634 (14)	0.0779 (17)	0.0781 (16)	−0.0121 (13)	−0.0175 (13)	−0.0093 (14)
C7	0.0763 (16)	0.0815 (18)	0.0650 (14)	−0.0075 (14)	−0.0128 (13)	−0.0140 (13)
C7A	0.0575 (13)	0.0550 (14)	0.0593 (13)	0.0059 (11)	−0.0074 (10)	−0.0029 (11)
C21	0.0583 (12)	0.0519 (13)	0.0534 (12)	0.0042 (10)	0.0086 (9)	−0.0048 (10)
C22	0.0747 (15)	0.0627 (15)	0.0605 (14)	0.0115 (12)	0.0049 (11)	0.0077 (12)
C23	0.0854 (17)	0.0586 (15)	0.0639 (14)	0.0042 (13)	0.0178 (12)	0.0109 (12)
C23A	0.0712 (14)	0.0465 (12)	0.0576 (13)	−0.0023 (11)	0.0214 (11)	−0.0037 (10)
C24	0.0880 (18)	0.0624 (16)	0.0828 (17)	−0.0119 (14)	0.0351 (15)	0.0031 (13)
C25	0.0704 (16)	0.0733 (17)	0.0985 (19)	−0.0165 (14)	0.0335 (15)	−0.0126 (16)
C25A	0.0564 (13)	0.0593 (14)	0.0728 (15)	−0.0020 (11)	0.0152 (11)	−0.0223 (12)
C26	0.0588 (14)	0.0823 (19)	0.0951 (19)	−0.0004 (13)	0.0096 (14)	−0.0338 (16)
C27	0.0648 (16)	0.093 (2)	0.0861 (19)	0.0166 (15)	−0.0078 (13)	−0.0238 (17)
C28	0.0741 (16)	0.0770 (17)	0.0732 (16)	0.0168 (14)	−0.0005 (12)	−0.0047 (13)
C28A	0.0603 (13)	0.0567 (13)	0.0555 (12)	0.0068 (11)	0.0054 (10)	−0.0077 (11)
C29	0.0738 (15)	0.0660 (15)	0.0625 (14)	0.0041 (13)	0.0100 (12)	0.0122 (12)
C210	0.0570 (13)	0.0623 (15)	0.0635 (13)	−0.0041 (11)	0.0111 (10)	0.0076 (11)
C20A	0.0569 (12)	0.0446 (11)	0.0461 (11)	0.0002 (10)	0.0107 (9)	−0.0034 (9)
C23B	0.0579 (12)	0.0415 (11)	0.0499 (11)	0.0004 (9)	0.0141 (9)	−0.0104 (9)
C25B	0.0557 (12)	0.0476 (12)	0.0526 (12)	0.0012 (10)	0.0115 (9)	−0.0142 (10)
C11	0.0969 (18)	0.0789 (18)	0.0503 (13)	0.0105 (15)	0.0062 (12)	−0.0079 (12)
C12	0.077 (3)	0.065 (3)	0.062 (2)	0.011 (2)	0.017 (2)	0.000 (2)
C13	0.175 (13)	0.073 (6)	0.113 (6)	0.030 (7)	0.053 (6)	0.014 (6)
C14	0.0969 (18)	0.0789 (18)	0.0503 (13)	0.0105 (15)	0.0062 (12)	−0.0079 (12)
C15	0.196 (14)	0.093 (8)	0.095 (6)	0.012 (8)	0.061 (7)	−0.028 (6)
C16	0.118 (10)	0.066 (8)	0.170 (12)	0.022 (8)	0.069 (9)	−0.016 (8)
C51	0.0540 (13)	0.0631 (15)	0.0888 (18)	−0.0051 (12)	−0.0090 (12)	0.0000 (14)
O51	0.0718 (11)	0.1071 (15)	0.1238 (15)	−0.0365 (11)	−0.0019 (10)	−0.0166 (12)
O52	0.0664 (10)	0.1052 (14)	0.0885 (12)	−0.0267 (10)	0.0129 (9)	−0.0138 (11)
C52	0.0686 (16)	0.111 (2)	0.0919 (19)	−0.0163 (16)	0.0125 (14)	0.0105 (17)
C53	0.129 (3)	0.100 (2)	0.138 (3)	0.023 (2)	0.051 (2)	0.017 (2)

### Ethyl 1-propyl-2-(pyren-1-yl)-1H-benzimidazole-5-carboxylate (II) Geometric parameters (Å, º)

**Table:**

N1—C7A	1.375 (3)	C28—C28A	1.399 (3)
N1—C2	1.386 (3)	C28—H28	0.9300
N1—C11	1.472 (3)	C28A—C25B	1.413 (3)
C2—N3	1.315 (3)	C28A—C29	1.426 (3)
C2—C21	1.477 (3)	C29—C210	1.342 (3)
N3—C3A	1.383 (2)	C29—H29	0.9300
C3A—C4	1.385 (3)	C210—C20A	1.434 (3)
C3A—C7A	1.402 (3)	C210—H210	0.9300
C4—C5	1.381 (3)	C20A—C23B	1.421 (3)
C4—H4	0.9300	C23B—C25B	1.424 (3)
C5—C6	1.404 (3)	C11—C12	1.516 (4)
C5—C51	1.484 (3)	C11—H11A	0.9700
C6—C7	1.368 (3)	C11—H11B	0.9700
C6—H6	0.9300	C12—C13	1.526 (10)
C7—C7A	1.394 (3)	C12—H12A	0.9700
C7—H7	0.9300	C12—H12B	0.9700
C21—C22	1.392 (3)	C13—H13A	0.9600
C21—C20A	1.408 (3)	C13—H13B	0.9600
C22—C23	1.372 (3)	C13—H13C	0.9600
C22—H22	0.9300	C15—C16	1.529 (12)
C23—C23A	1.388 (3)	C15—H15A	0.9700
C23—H23	0.9300	C15—H15B	0.9700
C23A—C23B	1.421 (3)	C16—H16A	0.9600
C23A—C24	1.436 (3)	C16—H16B	0.9600
C24—C25	1.320 (3)	C16—H16C	0.9600
C24—H24	0.9300	C51—O51	1.202 (3)
C25—C25A	1.431 (3)	C51—O52	1.324 (3)
C25—H25	0.9300	O52—C52	1.459 (3)
C25A—C26	1.390 (3)	C52—C53	1.474 (4)
C25A—C25B	1.417 (3)	C52—H52A	0.9700
C26—C27	1.370 (4)	C52—H52B	0.9700
C26—H26	0.9300	C53—H53A	0.9600
C27—C28	1.369 (3)	C53—H53B	0.9600
C27—H27	0.9300	C53—H53C	0.9600
			
C7A—N1—C2	105.76 (17)	C210—C29—C28A	122.2 (2)
C7A—N1—C11	125.70 (18)	C210—C29—H29	118.9
C2—N1—C11	128.37 (19)	C28A—C29—H29	118.9
N3—C2—N1	113.17 (18)	C29—C210—C20A	121.5 (2)
N3—C2—C21	124.54 (18)	C29—C210—H210	119.2
N1—C2—C21	122.28 (18)	C20A—C210—H210	119.2
C2—N3—C3A	104.99 (17)	C21—C20A—C23B	118.70 (18)
N3—C3A—C4	129.65 (19)	C21—C20A—C210	123.67 (19)
N3—C3A—C7A	109.96 (18)	C23B—C20A—C210	117.62 (18)
C4—C3A—C7A	120.38 (19)	C20A—C23B—C23A	120.15 (18)
C5—C4—C3A	118.5 (2)	C20A—C23B—C25B	120.52 (18)
C5—C4—H4	120.7	C23A—C23B—C25B	119.32 (18)
C3A—C4—H4	120.7	C28A—C25B—C25A	119.50 (19)
C4—C5—C6	120.1 (2)	C28A—C25B—C23B	119.98 (18)
C4—C5—C51	121.2 (2)	C25A—C25B—C23B	120.5 (2)
C6—C5—C51	118.7 (2)	N1—C11—C12	115.1 (2)
C7—C6—C5	122.6 (2)	N1—C11—H11A	108.5
C7—C6—H6	118.7	C12—C11—H11A	108.5
C5—C6—H6	118.7	N1—C11—H11B	108.5
C6—C7—C7A	116.8 (2)	C12—C11—H11B	108.5
C6—C7—H7	121.6	H11A—C11—H11B	107.5
C7A—C7—H7	121.6	C11—C12—C13	111.4 (7)
N1—C7A—C7	132.3 (2)	C11—C12—H12A	109.3
N1—C7A—C3A	106.11 (17)	C13—C12—H12A	109.3
C7—C7A—C3A	121.6 (2)	C11—C12—H12B	109.3
C22—C21—C20A	119.75 (19)	C13—C12—H12B	109.3
C22—C21—C2	119.41 (19)	H12A—C12—H12B	108.0
C20A—C21—C2	120.84 (19)	C12—C13—H13A	109.5
C23—C22—C21	121.5 (2)	C12—C13—H13B	109.5
C23—C22—H22	119.3	H13A—C13—H13B	109.5
C21—C22—H22	119.3	C12—C13—H13C	109.5
C22—C23—C23A	120.9 (2)	H13A—C13—H13C	109.5
C22—C23—H23	119.5	H13B—C13—H13C	109.5
C23A—C23—H23	119.5	C16—C15—H15A	109.4
C23—C23A—C23B	119.0 (2)	C16—C15—H15B	109.4
C23—C23A—C24	122.7 (2)	H15A—C15—H15B	108.0
C23B—C23A—C24	118.3 (2)	C15—C16—H16A	109.5
C25—C24—C23A	121.8 (2)	C15—C16—H16B	109.5
C25—C24—H24	119.1	H16A—C16—H16B	109.5
C23A—C24—H24	119.1	C15—C16—H16C	109.5
C24—C25—C25A	122.3 (2)	H16A—C16—H16C	109.5
C24—C25—H25	118.9	H16B—C16—H16C	109.5
C25A—C25—H25	118.9	O51—C51—O52	122.4 (2)
C26—C25A—C25B	119.1 (2)	O51—C51—C5	124.2 (2)
C26—C25A—C25	123.1 (2)	O52—C51—C5	113.46 (19)
C25B—C25A—C25	117.8 (2)	C51—O52—C52	117.52 (19)
C27—C26—C25A	120.7 (2)	O52—C52—C53	111.6 (2)
C27—C26—H26	119.7	O52—C52—H52A	109.3
C25A—C26—H26	119.7	C53—C52—H52A	109.3
C28—C27—C26	121.2 (2)	O52—C52—H52B	109.3
C28—C27—H27	119.4	C53—C52—H52B	109.3
C26—C27—H27	119.4	H52A—C52—H52B	108.0
C27—C28—C28A	120.5 (2)	C52—C53—H53A	109.5
C27—C28—H28	119.7	C52—C53—H53B	109.5
C28A—C28—H28	119.7	H53A—C53—H53B	109.5
C28—C28A—C25B	119.0 (2)	C52—C53—H53C	109.5
C28—C28A—C29	122.9 (2)	H53A—C53—H53C	109.5
C25B—C28A—C29	118.15 (19)	H53B—C53—H53C	109.5
			
C7A—N1—C2—N3	−0.8 (2)	C26—C27—C28—C28A	−0.7 (4)
C11—N1—C2—N3	−176.1 (2)	C27—C28—C28A—C25B	0.7 (3)
C7A—N1—C2—C21	−179.88 (19)	C27—C28—C28A—C29	−178.9 (2)
C11—N1—C2—C21	4.7 (3)	C28—C28A—C29—C210	179.2 (2)
N1—C2—N3—C3A	0.3 (2)	C25B—C28A—C29—C210	−0.4 (3)
C21—C2—N3—C3A	179.40 (19)	C28A—C29—C210—C20A	0.3 (4)
C2—N3—C3A—C4	178.9 (2)	C22—C21—C20A—C23B	−1.4 (3)
C2—N3—C3A—C7A	0.3 (2)	C2—C21—C20A—C23B	179.00 (17)
N3—C3A—C4—C5	−177.3 (2)	C22—C21—C20A—C210	179.4 (2)
C7A—C3A—C4—C5	1.2 (3)	C2—C21—C20A—C210	−0.2 (3)
C3A—C4—C5—C6	−1.3 (3)	C29—C210—C20A—C21	179.6 (2)
C3A—C4—C5—C51	177.2 (2)	C29—C210—C20A—C23B	0.4 (3)
C4—C5—C6—C7	0.6 (4)	C21—C20A—C23B—C23A	0.5 (3)
C51—C5—C6—C7	−178.0 (2)	C210—C20A—C23B—C23A	179.80 (18)
C5—C6—C7—C7A	0.4 (4)	C21—C20A—C23B—C25B	179.80 (18)
C2—N1—C7A—C7	−178.2 (2)	C210—C20A—C23B—C25B	−0.9 (3)
C11—N1—C7A—C7	−2.7 (4)	C23—C23A—C23B—C20A	0.6 (3)
C2—N1—C7A—C3A	0.9 (2)	C24—C23A—C23B—C20A	179.60 (18)
C11—N1—C7A—C3A	176.41 (19)	C23—C23A—C23B—C25B	−178.71 (19)
C6—C7—C7A—N1	178.5 (2)	C24—C23A—C23B—C25B	0.3 (3)
C6—C7—C7A—C3A	−0.5 (3)	C28—C28A—C25B—C25A	−0.2 (3)
N3—C3A—C7A—N1	−0.7 (2)	C29—C28A—C25B—C25A	179.43 (18)
C4—C3A—C7A—N1	−179.50 (19)	C28—C28A—C25B—C23B	−179.73 (19)
N3—C3A—C7A—C7	178.5 (2)	C29—C28A—C25B—C23B	−0.1 (3)
C4—C3A—C7A—C7	−0.3 (3)	C26—C25A—C25B—C28A	−0.3 (3)
N3—C2—C21—C22	−120.0 (2)	C25—C25A—C25B—C28A	179.22 (19)
N1—C2—C21—C22	59.0 (3)	C26—C25A—C25B—C23B	179.24 (19)
N3—C2—C21—C20A	59.6 (3)	C25—C25A—C25B—C23B	−1.2 (3)
N1—C2—C21—C20A	−121.4 (2)	C20A—C23B—C25B—C28A	0.8 (3)
C20A—C21—C22—C23	1.1 (3)	C23A—C23B—C25B—C28A	−179.91 (18)
C2—C21—C22—C23	−179.3 (2)	C20A—C23B—C25B—C25A	−178.75 (17)
C21—C22—C23—C23A	0.0 (3)	C23A—C23B—C25B—C25A	0.5 (3)
C22—C23—C23A—C23B	−0.9 (3)	C7A—N1—C11—C12	−112.6 (3)
C22—C23—C23A—C24	−179.9 (2)	C2—N1—C11—C12	61.9 (4)
C23—C23A—C24—C25	178.5 (2)	N1—C11—C12—C13	59.1 (9)
C23B—C23A—C24—C25	−0.5 (3)	C4—C5—C51—O51	−173.1 (2)
C23A—C24—C25—C25A	−0.2 (4)	C6—C5—C51—O51	5.5 (4)
C24—C25—C25A—C26	−179.4 (2)	C4—C5—C51—O52	5.7 (3)
C24—C25—C25A—C25B	1.1 (3)	C6—C5—C51—O52	−175.8 (2)
C25B—C25A—C26—C27	0.3 (3)	O51—C51—O52—C52	1.8 (4)
C25—C25A—C26—C27	−179.2 (2)	C5—C51—O52—C52	−177.0 (2)
C25A—C26—C27—C28	0.1 (4)	C51—O52—C52—C53	−83.7 (3)

### Ethyl 1-propyl-2-(pyren-1-yl)-1H-benzimidazole-5-carboxylate (II) Hydrogen-bond geometry (Å, º)

**Table:**

*D*—H···*A*	*D*—H	H···*A*	*D*···*A*	*D*—H···*A*
C11—H11*A*···O51^i^	0.97	2.51	3.253 (3)	133
C22—H22···O51^i^	0.93	2.37	3.290 (3)	168

Symmetry code: (i) −*x*+1, *y*+1/2, −*z*+1/2.

### Ethyl 1-methyl-2-(5-chloro-3-methyl-1-phenyl-1H-pyrazol-4-yl)-1H-benzimidazole-5-carboxylate (III) Crystal data

**Table:**

C_21_H_19_ClN_4_O_2_	*F*(000) = 824
*M**_r_* = 394.85	*D*_x_ = 1.334 Mg m^−^^3^
Monoclinic, *P*2_1_/*n*	Mo *K*α radiation, λ = 0.71073 Å
*a* = 11.1095 (4) Å	Cell parameters from 4250 reflections
*b* = 9.5126 (4) Å	θ = 2.8–27.8°
*c* = 18.6747 (8) Å	µ = 0.22 mm^−^^1^
β = 95.079 (4)°	*T* = 296 K
*V* = 1965.80 (14) Å^3^	Block, yellow
*Z* = 4	0.48 × 0.40 × 0.40 mm

### Ethyl 1-methyl-2-(5-chloro-3-methyl-1-phenyl-1H-pyrazol-4-yl)-1H-benzimidazole-5-carboxylate (III) Data collection

**Table:**

Oxford Diffraction Xcalibur with Sapphire CCD diffractometer	4250 independent reflections
Radiation source: Enhance (Mo) X-ray Source	3323 reflections with *I* > 2σ(*I*)
Graphite monochromator	*R*_int_ = 0.013
ω scans	θ_max_ = 27.8°, θ_min_ = 2.8°
Absorption correction: multi-scan (CrysAlis RED; Oxford Diffraction, 2009)	*h* = −14→14
*T*_min_ = 0.808, *T*_max_ = 0.916	*k* = −12→10
8067 measured reflections	*l* = −20→24

### Ethyl 1-methyl-2-(5-chloro-3-methyl-1-phenyl-1H-pyrazol-4-yl)-1H-benzimidazole-5-carboxylate (III) Refinement

**Table:**

Refinement on *F*^2^	Primary atom site location: difference Fourier map
Least-squares matrix: full	Hydrogen site location: inferred from neighbouring sites
*R*[*F*^2^ > 2σ(*F*^2^)] = 0.040	H-atom parameters constrained
*wR*(*F*^2^) = 0.109	*w* = 1/[σ^2^(*F*_o_^2^) + (0.0547*P*)^2^ + 0.5825*P*] where *P* = (*F*_o_^2^ + 2*F*_c_^2^)/3
*S* = 1.02	(Δ/σ)_max_ = 0.001
4250 reflections	Δρ_max_ = 0.32 e Å^−^^3^
294 parameters	Δρ_min_ = −0.29 e Å^−^^3^
28 restraints	

### Ethyl 1-methyl-2-(5-chloro-3-methyl-1-phenyl-1H-pyrazol-4-yl)-1H-benzimidazole-5-carboxylate (III) Special details

**Table:**

Geometry. All esds (except the esd in the dihedral angle between two l.s. planes) are estimated using the full covariance matrix. The cell esds are taken into account individually in the estimation of esds in distances, angles and torsion angles; correlations between esds in cell parameters are only used when they are defined by crystal symmetry. An approximate (isotropic) treatment of cell esds is used for estimating esds involving l.s. planes.

### Ethyl 1-methyl-2-(5-chloro-3-methyl-1-phenyl-1H-pyrazol-4-yl)-1H-benzimidazole-5-carboxylate (III) Fractional atomic coordinates and isotropic or equivalent isotropic displacement parameters (Å^2^)

**Table:**

	*x*	*y*	*z*	*U*_iso_*/*U*_eq_	Occ. (<1)
N1	0.68249 (11)	0.22101 (15)	0.52839 (7)	0.0373 (3)	
C2	0.67704 (14)	0.25652 (17)	0.59966 (8)	0.0369 (3)	
N3	0.56674 (12)	0.25655 (16)	0.61981 (7)	0.0423 (3)	
C3A	0.49392 (14)	0.22047 (17)	0.55799 (8)	0.0367 (3)	
C4	0.36951 (14)	0.20581 (18)	0.54660 (9)	0.0405 (4)	
H4	0.3205	0.2204	0.5838	0.049*	
C5	0.31969 (14)	0.16872 (18)	0.47826 (9)	0.0391 (4)	
C6	0.39284 (15)	0.14650 (19)	0.42181 (9)	0.0429 (4)	
H6	0.3572	0.1214	0.3767	0.051*	
C7	0.51670 (15)	0.1612 (2)	0.43215 (8)	0.0431 (4)	
H7	0.5656	0.1467	0.3949	0.052*	
C7A	0.56520 (13)	0.19862 (17)	0.50056 (8)	0.0358 (3)	
C11	0.78783 (15)	0.1939 (2)	0.48958 (9)	0.0458 (4)	
H11A	0.8021	0.2731	0.4596	0.069*	
H11B	0.7739	0.1117	0.4602	0.069*	
H11C	0.8571	0.1790	0.5233	0.069*	
N21	0.95909 (11)	0.39053 (14)	0.68756 (7)	0.0368 (3)	
N22	0.92990 (12)	0.30048 (15)	0.74036 (7)	0.0404 (3)	
C23	0.82543 (14)	0.24234 (18)	0.71555 (8)	0.0388 (4)	
C24	0.78630 (14)	0.29305 (17)	0.64600 (8)	0.0362 (3)	
C25	0.87371 (14)	0.38756 (17)	0.63130 (8)	0.0365 (3)	
Cl25	0.87732 (4)	0.50148 (5)	0.56105 (2)	0.05466 (15)	
C211	1.07114 (14)	0.46565 (17)	0.69408 (8)	0.0380 (4)	
C212	1.15160 (15)	0.44554 (19)	0.64275 (9)	0.0444 (4)	
H212	1.1336	0.3831	0.6050	0.053*	
C213	1.25950 (17)	0.5195 (2)	0.64814 (11)	0.0557 (5)	
H213	1.3144	0.5067	0.6139	0.067*	
C214	1.28538 (19)	0.6115 (3)	0.70383 (12)	0.0660 (6)	
H214	1.3575	0.6616	0.7072	0.079*	
C215	1.2046 (2)	0.6295 (3)	0.75463 (12)	0.0740 (7)	
H215	1.2228	0.6918	0.7924	0.089*	
C216	1.09661 (18)	0.5567 (2)	0.75048 (10)	0.0572 (5)	
H216	1.0423	0.5691	0.7851	0.069*	
C231	0.76612 (18)	0.1354 (2)	0.75887 (10)	0.0566 (5)	
H31A	0.7397	0.0577	0.7286	0.085*	
H31C	0.6978	0.1769	0.7788	0.085*	
H31B	0.8227	0.1026	0.7971	0.085*	
C51	0.18619 (16)	0.1561 (2)	0.46711 (10)	0.0466 (4)	
O51	0.1166 (12)	0.1806 (17)	0.5157 (6)	0.0532 (18)	0.645 (7)
O52	0.1484 (4)	0.1283 (5)	0.4013 (3)	0.0534 (11)	0.645 (7)
C52	0.0170 (3)	0.1216 (6)	0.3863 (2)	0.0619 (12)	0.645 (7)
H52A	−0.0173	0.0617	0.4213	0.074*	0.645 (7)
H52B	−0.0175	0.2148	0.3895	0.074*	0.645 (7)
C53	−0.0108 (4)	0.0652 (7)	0.3143 (3)	0.101 (2)	0.645 (7)
H53A	0.0294	0.1201	0.2805	0.151*	0.645 (7)
H53B	−0.0965	0.0687	0.3021	0.151*	0.645 (7)
H53C	0.0163	−0.0305	0.3130	0.151*	0.645 (7)
O61	0.119 (2)	0.188 (3)	0.5021 (11)	0.054 (3)	0.355 (7)
O62	0.1531 (6)	0.0711 (9)	0.4014 (6)	0.0539 (19)	0.355 (7)
C62	0.0248 (6)	0.0360 (9)	0.3824 (4)	0.0618 (19)	0.355 (7)
H62A	0.0177	−0.0526	0.3567	0.074*	0.355 (7)
H62B	−0.0178	0.0283	0.4254	0.074*	0.355 (7)
C63	−0.0251 (7)	0.1499 (9)	0.3370 (6)	0.083 (3)	0.355 (7)
H63A	−0.1071	0.1282	0.3200	0.125*	0.355 (7)
H63B	0.0221	0.1613	0.2968	0.125*	0.355 (7)
H63C	−0.0233	0.2355	0.3644	0.125*	0.355 (7)

### Ethyl 1-methyl-2-(5-chloro-3-methyl-1-phenyl-1H-pyrazol-4-yl)-1H-benzimidazole-5-carboxylate (III) Atomic displacement parameters (Å^2^)

**Table:**

	*U*^11^	*U*^22^	*U*^33^	*U*^12^	*U*^13^	*U*^23^
N1	0.0295 (6)	0.0475 (8)	0.0354 (7)	0.0004 (6)	0.0057 (5)	−0.0019 (6)
C2	0.0349 (8)	0.0412 (9)	0.0347 (8)	0.0008 (7)	0.0040 (6)	0.0013 (6)
N3	0.0351 (7)	0.0575 (9)	0.0348 (7)	−0.0004 (6)	0.0054 (5)	−0.0028 (6)
C3A	0.0343 (8)	0.0428 (9)	0.0336 (8)	0.0017 (7)	0.0058 (6)	−0.0001 (6)
C4	0.0337 (8)	0.0505 (10)	0.0384 (8)	0.0030 (7)	0.0093 (6)	0.0006 (7)
C5	0.0313 (8)	0.0450 (9)	0.0409 (8)	0.0020 (7)	0.0033 (6)	0.0042 (7)
C6	0.0388 (8)	0.0543 (10)	0.0353 (8)	−0.0015 (8)	0.0016 (7)	−0.0024 (7)
C7	0.0377 (8)	0.0589 (11)	0.0335 (8)	−0.0007 (8)	0.0080 (6)	−0.0040 (7)
C7A	0.0305 (8)	0.0412 (9)	0.0361 (8)	0.0011 (6)	0.0054 (6)	0.0013 (6)
C11	0.0346 (8)	0.0577 (11)	0.0466 (9)	0.0007 (8)	0.0123 (7)	−0.0047 (8)
N21	0.0356 (7)	0.0413 (7)	0.0330 (6)	−0.0028 (6)	0.0005 (5)	0.0028 (5)
N22	0.0424 (7)	0.0464 (8)	0.0324 (7)	−0.0024 (6)	0.0022 (5)	0.0051 (6)
C23	0.0376 (8)	0.0444 (9)	0.0349 (8)	0.0002 (7)	0.0061 (6)	0.0015 (7)
C24	0.0329 (8)	0.0412 (9)	0.0345 (8)	0.0010 (6)	0.0034 (6)	−0.0001 (6)
C25	0.0373 (8)	0.0388 (8)	0.0331 (8)	0.0012 (7)	0.0009 (6)	0.0028 (6)
Cl25	0.0594 (3)	0.0554 (3)	0.0473 (3)	−0.0094 (2)	−0.0065 (2)	0.0195 (2)
C211	0.0363 (8)	0.0398 (9)	0.0372 (8)	−0.0022 (7)	−0.0001 (6)	0.0010 (6)
C212	0.0433 (9)	0.0458 (9)	0.0443 (9)	−0.0010 (8)	0.0048 (7)	−0.0041 (7)
C213	0.0444 (10)	0.0655 (13)	0.0582 (11)	−0.0057 (9)	0.0104 (8)	0.0054 (10)
C214	0.0524 (12)	0.0774 (15)	0.0665 (13)	−0.0264 (11)	−0.0042 (10)	0.0033 (11)
C215	0.0748 (15)	0.0873 (17)	0.0589 (12)	−0.0327 (13)	−0.0006 (11)	−0.0247 (12)
C216	0.0562 (11)	0.0709 (13)	0.0447 (10)	−0.0107 (10)	0.0064 (8)	−0.0150 (9)
C231	0.0574 (11)	0.0665 (13)	0.0461 (10)	−0.0141 (10)	0.0060 (8)	0.0122 (9)
C51	0.0349 (9)	0.0557 (11)	0.0489 (10)	0.0006 (8)	0.0014 (8)	0.0053 (8)
O51	0.035 (2)	0.077 (3)	0.049 (3)	−0.0055 (19)	0.013 (2)	0.001 (3)
O52	0.0304 (12)	0.073 (3)	0.0555 (15)	−0.0031 (15)	−0.0027 (10)	−0.016 (2)
C52	0.0280 (14)	0.081 (3)	0.074 (2)	0.0052 (18)	−0.0104 (14)	−0.022 (2)
C53	0.056 (2)	0.126 (5)	0.113 (4)	0.014 (3)	−0.029 (2)	−0.062 (3)
O61	0.038 (4)	0.071 (5)	0.057 (7)	0.011 (4)	0.019 (4)	0.004 (5)
O62	0.036 (2)	0.076 (5)	0.049 (3)	−0.011 (3)	−0.0015 (19)	−0.007 (4)
C62	0.063 (4)	0.065 (4)	0.060 (4)	0.012 (3)	0.014 (3)	−0.010 (3)
C63	0.072 (5)	0.080 (6)	0.096 (7)	0.005 (4)	0.000 (5)	−0.003 (5)

### Ethyl 1-methyl-2-(5-chloro-3-methyl-1-phenyl-1H-pyrazol-4-yl)-1H-benzimidazole-5-carboxylate (III) Geometric parameters (Å, º)

**Table:**

N1—C7A	1.3761 (19)	C212—H212	0.9300
N1—C2	1.380 (2)	C213—C214	1.370 (3)
N1—C11	1.453 (2)	C213—H213	0.9300
C2—N3	1.313 (2)	C214—C215	1.373 (3)
C2—C24	1.469 (2)	C214—H214	0.9300
N3—C3A	1.393 (2)	C215—C216	1.381 (3)
C3A—C4	1.387 (2)	C215—H215	0.9300
C3A—C7A	1.404 (2)	C216—H216	0.9300
C4—C5	1.391 (2)	C231—H31A	0.9600
C4—H4	0.9300	C231—H31C	0.9600
C5—C6	1.403 (2)	C231—H31B	0.9600
C5—C51	1.484 (2)	C51—O61	1.080 (19)
C6—C7	1.380 (2)	C51—O51	1.265 (10)
C6—H6	0.9300	C51—O52	1.290 (6)
C7—C7A	1.388 (2)	C51—O62	1.488 (10)
C7—H7	0.9300	O52—C52	1.463 (4)
C11—H11A	0.9600	C52—C53	1.455 (5)
C11—H11B	0.9600	C52—H52A	0.9700
C11—H11C	0.9600	C52—H52B	0.9700
N21—C25	1.3521 (19)	C53—H53A	0.9600
N21—N22	1.3666 (18)	C53—H53B	0.9600
N21—C211	1.431 (2)	C53—H53C	0.9600
N22—C23	1.331 (2)	O62—C62	1.477 (7)
C23—C24	1.417 (2)	C62—C63	1.455 (7)
C23—C231	1.489 (2)	C62—H62A	0.9700
C24—C25	1.369 (2)	C62—H62B	0.9700
C25—Cl25	1.7047 (15)	C63—H63A	0.9600
C211—C216	1.374 (2)	C63—H63B	0.9600
C211—C212	1.381 (2)	C63—H63C	0.9600
C212—C213	1.386 (3)		
			
C7A—N1—C2	106.34 (12)	C214—C213—C212	120.10 (18)
C7A—N1—C11	124.17 (13)	C214—C213—H213	120.0
C2—N1—C11	129.10 (13)	C212—C213—H213	120.0
N3—C2—N1	113.46 (14)	C213—C214—C215	119.88 (19)
N3—C2—C24	125.07 (14)	C213—C214—H214	120.1
N1—C2—C24	121.45 (14)	C215—C214—H214	120.1
C2—N3—C3A	104.52 (13)	C214—C215—C216	121.06 (19)
C4—C3A—N3	130.53 (14)	C214—C215—H215	119.5
C4—C3A—C7A	119.33 (14)	C216—C215—H215	119.5
N3—C3A—C7A	110.14 (13)	C211—C216—C215	118.57 (18)
C3A—C4—C5	118.46 (14)	C211—C216—H216	120.7
C3A—C4—H4	120.8	C215—C216—H216	120.7
C5—C4—H4	120.8	C23—C231—H31A	109.5
C4—C5—C6	121.19 (14)	C23—C231—H31C	109.5
C4—C5—C51	117.43 (15)	H31A—C231—H31C	109.5
C6—C5—C51	121.37 (15)	C23—C231—H31B	109.5
C7—C6—C5	121.10 (15)	H31A—C231—H31B	109.5
C7—C6—H6	119.5	H31C—C231—H31B	109.5
C5—C6—H6	119.5	O51—C51—O52	123.5 (6)
C6—C7—C7A	117.12 (14)	O61—C51—C5	129.0 (13)
C6—C7—H7	121.4	O51—C51—C5	123.5 (6)
C7A—C7—H7	121.4	O52—C51—C5	112.8 (3)
N1—C7A—C7	131.66 (14)	O61—C51—O62	121.3 (13)
N1—C7A—C3A	105.54 (13)	C5—C51—O62	109.3 (3)
C7—C7A—C3A	122.80 (14)	C51—O52—C52	115.3 (4)
N1—C11—H11A	109.5	C53—C52—O52	108.7 (4)
N1—C11—H11B	109.5	C53—C52—H52A	110.0
H11A—C11—H11B	109.5	O52—C52—H52A	110.0
N1—C11—H11C	109.5	C53—C52—H52B	110.0
H11A—C11—H11C	109.5	O52—C52—H52B	110.0
H11B—C11—H11C	109.5	H52A—C52—H52B	108.3
C25—N21—N22	110.65 (12)	C52—C53—H53A	109.5
C25—N21—C211	128.44 (13)	C52—C53—H53B	109.5
N22—N21—C211	120.77 (12)	H53A—C53—H53B	109.5
C23—N22—N21	105.33 (12)	C52—C53—H53C	109.5
N22—C23—C24	111.40 (14)	H53A—C53—H53C	109.5
N22—C23—C231	120.57 (14)	H53B—C53—H53C	109.5
C24—C23—C231	128.01 (15)	C62—O62—C51	119.0 (7)
C25—C24—C23	104.00 (13)	C63—C62—O62	106.4 (6)
C25—C24—C2	126.95 (14)	C63—C62—H62A	110.5
C23—C24—C2	129.04 (15)	O62—C62—H62A	110.5
N21—C25—C24	108.61 (13)	C63—C62—H62B	110.5
N21—C25—Cl25	121.47 (12)	O62—C62—H62B	110.5
C24—C25—Cl25	129.59 (12)	H62A—C62—H62B	108.6
C216—C211—C212	121.17 (16)	C62—C63—H63A	109.5
C216—C211—N21	119.91 (15)	C62—C63—H63B	109.5
C212—C211—N21	118.91 (14)	H63A—C63—H63B	109.5
C211—C212—C213	119.22 (17)	C62—C63—H63C	109.5
C211—C212—H212	120.4	H63A—C63—H63C	109.5
C213—C212—H212	120.4	H63B—C63—H63C	109.5
			
C7A—N1—C2—N3	0.98 (19)	C211—N21—C25—C24	175.07 (15)
C11—N1—C2—N3	−171.98 (16)	N22—N21—C25—Cl25	173.36 (11)
C7A—N1—C2—C24	−177.73 (15)	C211—N21—C25—Cl25	−10.9 (2)
C11—N1—C2—C24	9.3 (3)	C23—C24—C25—N21	0.95 (18)
N1—C2—N3—C3A	−0.72 (19)	C2—C24—C25—N21	−179.91 (15)
C24—C2—N3—C3A	177.93 (16)	C23—C24—C25—Cl25	−172.42 (13)
C2—N3—C3A—C4	−179.16 (18)	C2—C24—C25—Cl25	6.7 (3)
C2—N3—C3A—C7A	0.20 (18)	C25—N21—C211—C216	124.20 (19)
N3—C3A—C4—C5	179.75 (17)	N22—N21—C211—C216	−60.5 (2)
C7A—C3A—C4—C5	0.4 (2)	C25—N21—C211—C212	−55.3 (2)
C3A—C4—C5—C6	0.0 (3)	N22—N21—C211—C212	120.02 (17)
C3A—C4—C5—C51	−178.95 (16)	C216—C211—C212—C213	−0.4 (3)
C4—C5—C6—C7	−0.2 (3)	N21—C211—C212—C213	179.05 (16)
C51—C5—C6—C7	178.64 (17)	C211—C212—C213—C214	−0.1 (3)
C5—C6—C7—C7A	0.1 (3)	C212—C213—C214—C215	0.5 (3)
C2—N1—C7A—C7	179.74 (18)	C213—C214—C215—C216	−0.3 (4)
C11—N1—C7A—C7	−6.9 (3)	C212—C211—C216—C215	0.6 (3)
C2—N1—C7A—C3A	−0.77 (17)	N21—C211—C216—C215	−178.90 (19)
C11—N1—C7A—C3A	172.62 (15)	C214—C215—C216—C211	−0.2 (4)
C6—C7—C7A—N1	179.79 (17)	C4—C5—C51—O61	11 (2)
C6—C7—C7A—C3A	0.4 (3)	C6—C5—C51—O61	−167 (2)
C4—C3A—C7A—N1	179.81 (15)	C4—C5—C51—O51	1.8 (10)
N3—C3A—C7A—N1	0.38 (18)	C6—C5—C51—O51	−177.1 (9)
C4—C3A—C7A—C7	−0.6 (3)	C4—C5—C51—O52	176.7 (3)
N3—C3A—C7A—C7	179.92 (16)	C6—C5—C51—O52	−2.2 (3)
C25—N21—N22—C23	0.02 (18)	C4—C5—C51—O62	−160.8 (4)
C211—N21—N22—C23	−176.07 (14)	C6—C5—C51—O62	20.3 (5)
N21—N22—C23—C24	0.60 (18)	O61—C51—O52—C52	−9.8 (19)
N21—N22—C23—C231	179.07 (16)	O51—C51—O52—C52	−2.1 (11)
N22—C23—C24—C25	−0.97 (19)	C5—C51—O52—C52	−177.1 (3)
C231—C23—C24—C25	−179.30 (18)	O62—C51—O52—C52	98.2 (16)
N22—C23—C24—C2	179.91 (16)	C51—O52—C52—C53	−169.9 (6)
C231—C23—C24—C2	1.6 (3)	O61—C51—O62—C62	3 (2)
N3—C2—C24—C25	−128.63 (19)	O51—C51—O62—C62	13.4 (13)
N1—C2—C24—C25	49.9 (2)	O52—C51—O62—C62	−80.8 (13)
N3—C2—C24—C23	50.3 (3)	C5—C51—O62—C62	175.7 (5)
N1—C2—C24—C23	−131.15 (18)	C51—O62—C62—C63	89.5 (11)
N22—N21—C25—C24	−0.65 (18)		

### Ethyl 1-methyl-2-(5-chloro-3-methyl-1-phenyl-1H-pyrazol-4-yl)-1H-benzimidazole-5-carboxylate (III) Hydrogen-bond geometry (Å, º)

**Table:**

*D*—H···*A*	*D*—H	H···*A*	*D*···*A*	*D*—H···*A*
C212—H212···O51^i^	0.93	2.54	3.460 (14)	168
C212—H212···O61^i^	0.93	2.67	3.59 (2)	170

Symmetry code: (i) *x*+1, *y*, *z*.
